# A flower-shaped dual-band four port MIMO antenna with novel DGS for 5G mmWave applications

**DOI:** 10.1371/journal.pone.0358640

**Published:** 2026-09-28

**Authors:** Mohammed Bendaoued, Anouar Es-saleh, Soufian Lakrit, Sudipta Das, Abdelhamid Bou-El-Harmel, Manish Varun Yadav, Boddapati Traka Phani Madhav, Heba G. Mohamed

**Affiliations:** 1 LMIET FST of Settat, University of Hassan, Settat, Casablanca-Settat, Morocco; 2 Physical Sciences and Technology Innovation Laboratory, Higher School of Technology of Nador, Mohammed First University, Oujda, Morocco; 3 Department of Electronics and Communication Engineering, IMPS College of Engineering & Technology, Malda, West Bengal, India; 4 Laboratory of Innovative Technologies and Computer Science, Higher School of Technology, Sidi Mohamed Ben Abdellah University, Fez, Morocco; 5 Manipal Institute of Technology, Manipal Academy of Higher Education, Manipal, India; 6 Department of Electronics and Communication Engineering, Koneru Lakshmaiah Education Foundation, Guntur, Andra Pradesh, India; 7 Department of Electrical Engineering, College of Engineering, Princess Nourah bint Abdulrahman University, Riyadh, Saudi Arabia; 8 Electrical Department, College of Engineering, Alexandria Higher Institute of Engineering and Technology, Alexandria, Egypt; Northwestern Polytechnical University, CHINA

## Abstract

A novel quad-port multiple-input multiple-output (MIMO) antenna with enhanced isolation is proposed by incorporating a specifically designed hybrid defected ground structure (DGS) for dual band 5G mmWave wireless applications. This suggested MIMO antenna system features a distinctive DGS, consisting of a central circular slot combined with four surrounding trapezoidal cut-outs at the base of the ground plane for enhanced isolation and comprises four identical elliptical monopole elements. Initially, a four-element MIMO system is developed to achieve optimal performance. Subsequently, the conFigd hybrid DGS is included in the proposed structure to improve the isolation by effectively analyzing the impact of alterations in the various geometrical parameters of the DGS. The designed MIMO radiator resonates at 26.73/38.29 GHz and operates from 26.39 to 27.04 GHz and from 38.11 to 38.47 GHz, achieving gains of 4.80 dBi and 7.61 dBi at 26.73 GHz and 38.29 GHz, respectively, maintaining a compact size of 21 mm × 21 mm (1.849 λ × 1.849 λ). The designed four-port MIMO radiator demonstrates exceptional performance in terms of various impedance, radiation and diversity metrics. The design and simulation studies are conducted using the CST simulation tool. Finally, the intended antenna has been prototyped and experimentally tested, confirming the correctness of the simulation analysis. The proposed antenna partially operates within 5G NR mmWave n258/n260 bands and is suitable for 5G Fixed Wireless Access (FWA) applications.

## 1. Introduction

Managing multiple antennas in the user equipment’s transmitter and/or reception is more complicated than operating a single, well-isolated antenna system. The 5G, or fifth-generation wireless communication networks, are dispersed widely and utilize technology that follows the 4G. The characteristics of MIMO antenna systems include reduced latency, increased data throughput, and improved isolation between antenna parts [1]. To achieve the unprecedented reliability, capacity, and speed that characterize the fifth generation of wireless communication, MIMO antennas are essential for 5G technology [2]. In 5G applications, the four-port MIMO antenna system is an essential component among other MIMO design configurations. By using four transmitting and receiving antennas, this setup allows 5G networks to improve their capacity and coverage with increased spatial diversity and multiplexing [3–6]. Four-port MIMO antennas can enhance five-generation wireless communication performance in several ways, including enhanced data rates, larger network capacity, and improved dependability [7,8]. The four-port MIMO antennas improve wireless performance by utilizing multiple transmission paths to provide faster data throughput, reliable signal connection, and better network coverage in complex propagation environments. These antennas also help minimize co and adjacent channel interference, which help for better utilization of spectrum contention in congested network [9–11]. Recent literature introduces innovative antenna configurations like dual-operating four-port MIMO systems to fulfill 5G demands for high speed, low latency, and reliability. This setup transmits and receives multiple data streams simultaneously, boosting transfer speed and network efficiency [12].

The performance of antennas can be enhanced by incorporating novel DGS configurations. DGS is a unique electromagnetic structure that helps mitigate interference, improve radiation characteristics, and reduce backward radiation. Specifically, it is critical and challenging to minimize interference and optimize signal quality in 5G applications, which makes DGS a valuable addition to the four-port MIMO antenna [13]. This innovative antenna configuration and DGS integration contribute to the successful implementation of 5G technology, particularly in scenarios where high-frequency bands are used to achieve ultra-fast data rates and support massive device connectivity. The inclusion of a novel defected ground structure (DGS) with MIMO antennas is a significant design step in optimizing their performance. The strategic design of DGS involves manipulating the electromagnetic properties of the ground plane beneath an antenna. By introducing defects or variations in the ground plane, DGS can achieve various objectives such as reducing interference, improving bandwidth, radiation patterns, radiation efficiency of an antenna. The combination of MIMO technology and DGS holds an enormous promise for 5G uses. It enables the reduction of co-channel interference and enhances the antenna’s capabilities in terms of signal quality, connectivity, and coverage, which are essential factors in realizing the potential of 5G networks for many different uses, including augmented reality, autonomous vehicle, and the Internet of Things [14,15].

MIMO antennas with DGS in the ground plane offer several advantages in 5G and other wireless applications:

- Improved Isolation or Reduced Mutual Coupling: DGS can help to minimize mutual coupling between radiating elements in a MIMO system, thus, enhancing isolation between channels and reducing interference.
- Enhanced Radiation Patterns: The use of DGS can shape the radiation patterns of MIMO antennas, allowing for better coverage and directionality as needed in different scenarios.
- Bandwidth Improvement: The implementation of DGS can extend the bandwidth of the antenna, enabling support for multiple frequency bands and diverse communication requirements.
- Interference Reduction: The DGS-based antenna is capable to mitigate co-channel interference, improving signal quality in dense network environments. These characteristics make MIMO antennas with DGS in the ground plane a valuable choice for 5G applications, as they enable better performance, coverage, and network efficiency [16,17].

The next-generation 5G networks require dual-band mmWave MIMO antennas to counteract severe path loss and compact form-factor constraints. By concurrently operating across dual operating frequency bands within the mmWave spectrum, these systems enable extreme data rates and enhance spatial multiplexing, within constrained physical footprint of mobile devices. In this context, researchers have adopted several MIMO antenna design methodologies [18–23] to support dual band mmWave 5G communications.

The stated objective of this research is to develop a miniature-sized four elements-based dual band MIMO antenna with an overall improved performance. A novel hybrid DGS has been incorporated into the proposed structure to enhance isolation and facilitate miniaturization. A detailed investigation has been conducted on the impact of this hybrid DGS on the performance improvement of the suggested four-port MIMO radiator system. The proposed antenna shows considerable performance in terms of its impedance, radiation, and diversity metrics across the entire dual operating bands. The proposed four-port MIMO antenna achieves impedance bandwidths of about 0.65 GHz (26.39–27.04 GHz) and 0.36 GHz (38.11–38.47 GHz), resonating at 26.73 GHz and 38.29 GHz. The reflection coefficient drops to −60.94 and −16.80 dB at 26.73 and 38.29 GHz, respectively. The gain is 4.80 dBi at 26.73 GHz and 7.61 dBi at 38.29 GHz; the maximum radiation efficiencies are about 97.61% and 95.45% for the first and second band, respectively. The suggested antenna shows an exceptional diversity performance with ECC ≤ 0.00028, DG ≈10 dB, TARC ≤−15.42 dB, CCL < 0.1, MEG <−3 dB, and Multiplexing efficiency > 91.1%.

The novel contribution of this study is the development of a four-port MIMO antenna that combines four identical orthogonally arranged flower-shaped elliptical patch elements with a specifically engineered and systematically optimized hybrid defected ground structure (DGS) to achieve enhanced isolation across two distinct mmWave operating bands. The proposed DGS configuration is uniquely designed by a central circular slot combined with four surrounding trapezoidal slots to effectively improve isolation between the radiating patches while preserving high gain and radiation efficiency performance. Furthermore, it offers a favorable combination of dual-resonant band functionality, high isolation, size miniaturization, reliable radiative performance as well as excellent diversity metrics, ensuring improved signal reliability and maximized data throughput. This integrated design approach distinguishes the proposed antenna from previously reported MIMO structures, making it a potential choice for dual band 5G mmWave communication applications.

The following point summarizes the novel aspects of the suggested work:

- **Compact size:** The four identical elliptical monopole elements, transformed into flower shaped with a novel hybrid defected ground plane occupies an area of only 21 mm × 21 mm (1.849 λ × 1.849 λ).
- **Hybrid DGS configuration:** A distinctive DGS, consisting of circular and trapezoidal shapes are strategically incorporated at the ground plane’s base to minimize mutual coupling, improving overall isolation. This helps minimize the electromagnetic interference, improving overall system reliability.
- **Dual-Resonance and stable radiation performance:** The designed four port MIMO radiator operates from 26.39–27.04 GHz and 38.11–38.47 GHz, partially covering the spectrums of 5G NR n258/n260 bands, yielding stable gains of 4.80 and 7.61 dBi at 26.73 and 38.29 GHz, respectively along with radiation efficiencies of 97.61% and 95.45%, respectively.
- **Optimized metrics:** Exceptionally low ECC, high DG, competitive CCL, MEG, TARC and desired multiplexing efficiency confirm its superior MIMO diversity performance.
- **Performance validation and applicability:** All the analyzed results obtained by using CST MWS tool are validated through measurements of the realized antenna prototype. The measurement results confirm its dual band utilization. The proposed antenna partially operates within 5G NR mmWave n258/n260 bands and is suitable for 5G Fixed Wireless Access (FWA) applications.

## 2. Antenna layout with a unit patch

The suggested structure for the elemental antenna is displayed in [24], based on which two port and four port MIMO configurations are designed. A 50 Ω micro strip line feeds the antenna, which has a 12.5 × 6 mm^2^ partial ground plane. It is modelled on a 14 × 6 × 0.2 mm³ Rogers RO4003C dielectric slab with a permittivity of 3.55. The single antenna element is optimized and simulated by CST Microwave Studio. The recommended dual-band antenna’s ideal design specifications are pointed out in Table 1, and Fig 1 depicts its recommended geometry. The elliptical slot has five ellipses with an area, π × R_3_ × R_4_ = 1.34 mm^2^, while the patch has five ellipses with an area of π × R_1_ × R_2_ = 5.10 mm^2^. The modified patch shows a noticeable improvement in terms of gain, operational bandwidth, and reflection coefficient, as compared to basic patch. The patch antenna’s bandwidth is increased, and its size is miniaturized by using the specified slots. Fig 2 represents the fluctuations of the antenna’s (single patch) reflection coefficient vs. frequency. The patch antenna’s reflection coefficients display −18.39 dB at 26.76 GHz and an impedance bandwidth (IBW) of 0.48 GHz (26.52 to 27 GHz), and −46.89 dB at 38 GHz with an IBW of 0.43 GHz, which spans over the spectrum of 37.78 to 38.21 GHz, supporting two crucial 27/38 GHz 5G bands.

**Table 1:** The suggested antenna’s design specifications.

Characteristics	Values [mm]
L_G_	12.5
W_G_ = W_SUB_	6
H_G_ = H_F_	0.035
L_SUB_	14
L_F_	7.6
W_F_	0.54
R_1_	0.5
R_2_	3.25
R_3_	0.33
R_4_	1.3

**Fig 1:**
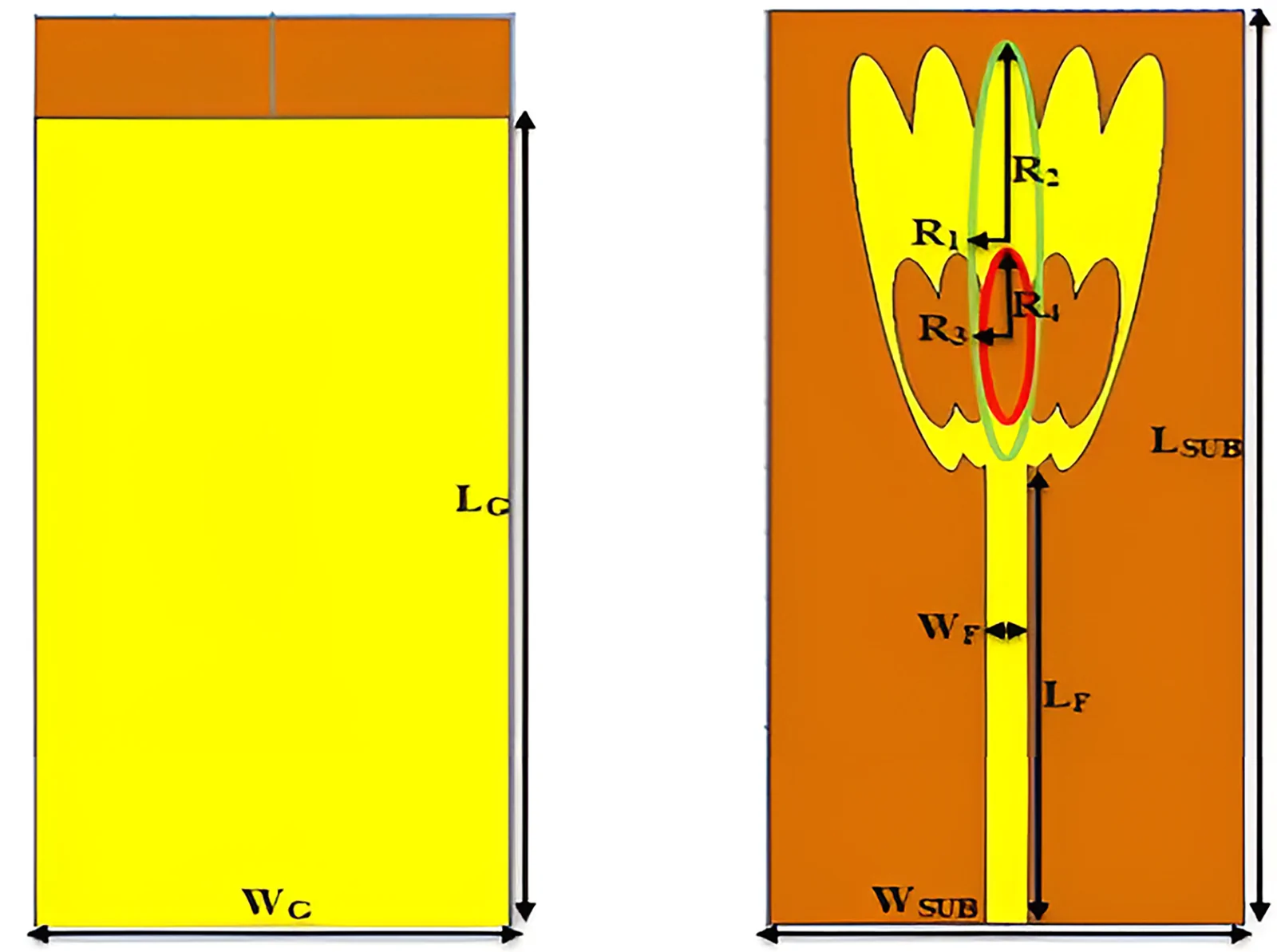
Configuration of the single-element radiator: the ground plane’s geometry, geometry of the patch.

**Fig 2:**
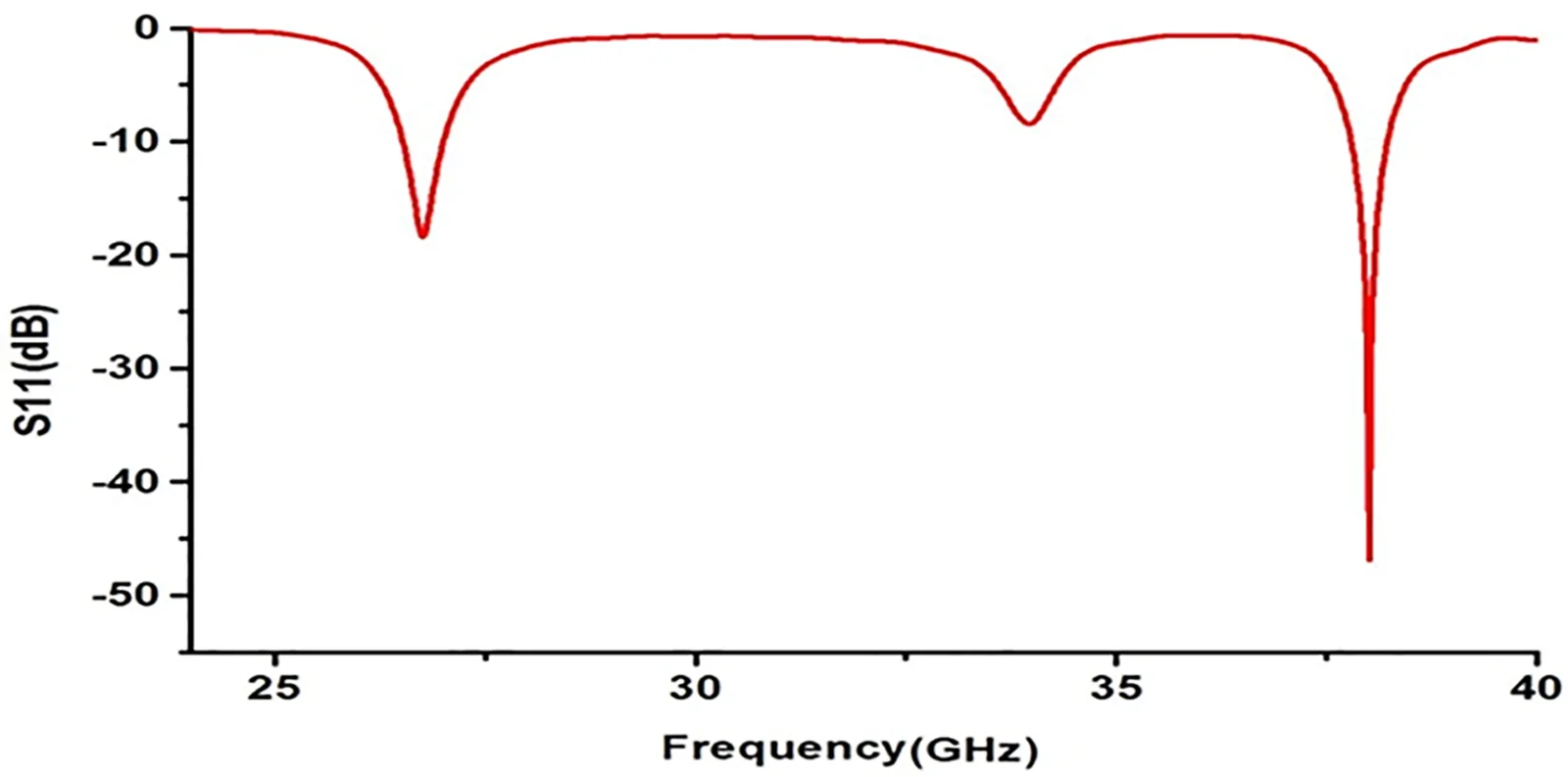
The proposed single antenna’s reflection coefficient in relation to frequencies.

## 3. Two-element MIMO antenna: Design perspectives and analysis of structures

Despite providing dual-band resonance with adequate impedance matching, this single-element design restricts operations to single-input-single-output (SISO), which limits data throughput and makes the system susceptible to multipath fading. Consequently, it is inadequate for high-data-rate 5G applications. In contrast, a two-element MIMO antenna deploys two independent radiators to handle multiple data streams simultaneously, effectively doubling capacity and dramatically improving signal reliability. The two-port setup provides a primary foundation for MIMO systems, enabling researchers to quantify mutual coupling, signal correlation, and overarching radiation behavior between antenna elements. Therefore, a two-element MIMO system is designed using two identical unit patch elements. Fig 3 displays four different configurations of the designed two-element MIMO antennas, which are developed by positioning the single-element antenna in various orientations.

**Fig 3:**
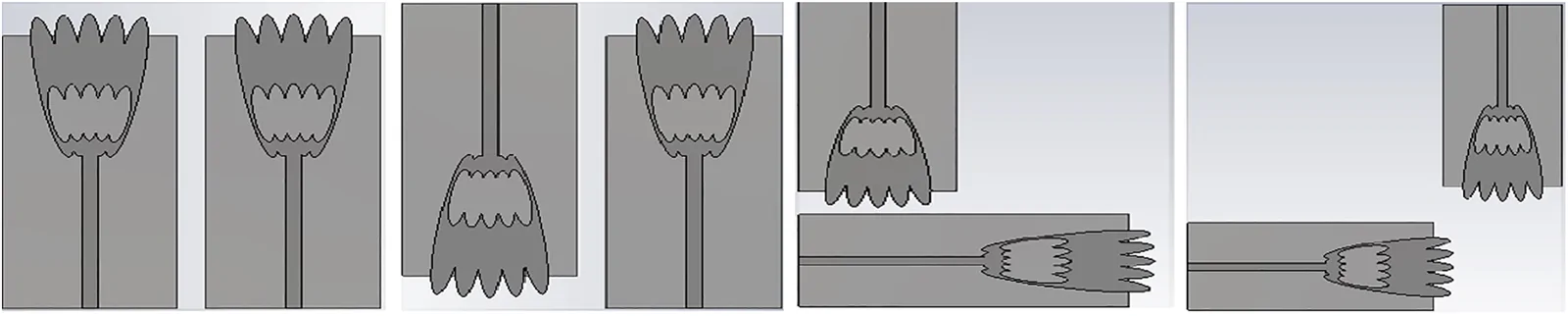
Design layout of two-port MIMO antenna showing placements of two elements in several positions, Two parallel elements, Two parallel opposing elements, Two perpendicular patch elements (case 1), and Two perpendicular elements (case 2).

These configurations are evaluated based on their reflection coefficients (S_11_) and isolation characteristics to identify the most suitable design. Fig 4 shows the S_11_ results: The two parallel elements configuration with a partial ground plane achieves S_11_ of −18 dB at 26.24 GHz along with an IBW of 0.46 GHz (26.02–26.48 GHz). For the second band, S_11_ is about −22.43 dB at 37.82 GHz, offering IBW of 0.35 GHz (37.65–38.0 GHz). The two perpendicular elements (case 2) also performs well, showing −18 dB at 26.2 GHz (bandwidth 0.47 GHz) and −16 dB at 37.7 GHz (bandwidth 0.29 GHz). In terms of isolation, both two perpendicular patch elements (case 1 and case 2) configurations demonstrate acceptable values below −15 dB, which is the typical threshold for MIMO systems. In conclusion, the configuration with two perpendicular patch elements (case 1) shows best impedance matching and isolation performance. Therefore, the configuration of two perpendicular patch elements (case 1) is selected as the proposed structure, due to its balanced performance in terms of reflection, isolation, and miniaturization.

**Fig 4:**
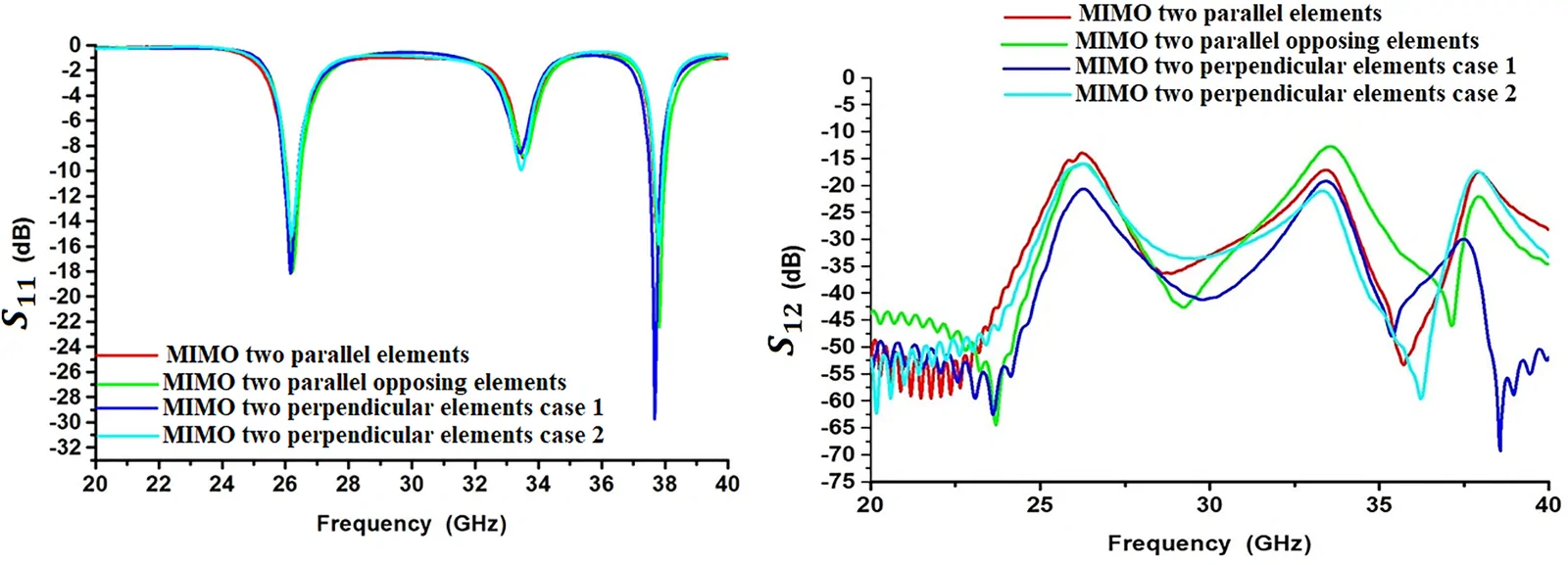
Two-element MIMO in different arrangements: reflection coefficient (S_11_) and isolation (S_12_).

## 4. Proposed four-element configuration of the MIMO antenna

This section investigates the four-element configuration of the MIMO antenna. The four-elemental radiating arrangement yields significantly higher data throughput**,** vastly improved signal reliability, and stronger link stability—especially intended for modern 5G networks. The configuration of the suggested MIMO radiator, a detailed analysis of the selection of intended ground plane geometry, and the impacts of various defected ground plane (DGS) design parameters are analyzed and discussed in this section.

### 4.1. Configuration of the antenna

A four-element MIMO radiator with the suggested design is outlined in Fig 5. It is constructed using a Rogers RO4003C substrate of 0.2 mm thickness, a loss tangent of 0.0025, and a permittivity of 3.55. The four elliptical flower-shaped radiating elements in the MIMO configuration are identical, and a novel “hybrid DGS” element is implemented for isolation enhancement. The total dimension of this compact MIMO antenna is 21 × 21 mm^2^. The proposed four-port MIMO antenna has an electrical size of 1.849 λ × 1.849 λ. The various essential optimal design parameters are summarized in Table 2.

**Table 2:** Physical parameters of the four-port MIMO radiator.

Characteristics	Values (mm)
W_SUB =_ L_SUB_	21
L_G_	12.5
W_G_	6
R_DGS_	2
h_Q_	2
H_Q_	2.5
W_Q_	5.5

**Fig 5:**
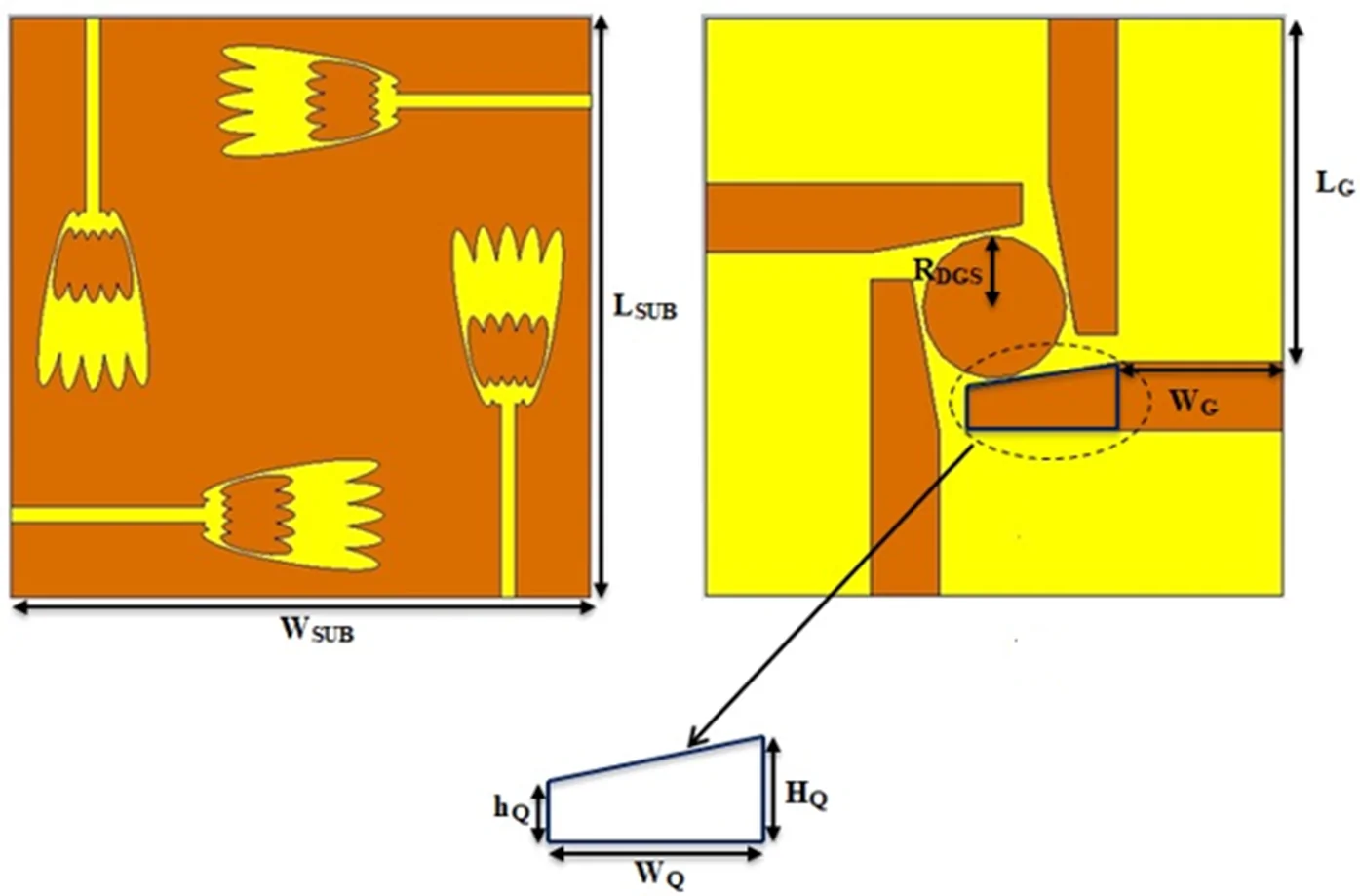
Geometry of the proposed MIMO antenna: geometry of four patch elements, partial ground plane with DGS, and Projection of DGS trapezoidal section.

### 4.2. Selection of ground plane with defected structure

To get optimal performance, the MIMO antenna is initially designed with four elements with ground planes. Secondly, a 15 × 15 mm^2^ square is used to connect the ground plane, and for greater isolation and miniaturization, the ground plane is made to be defected, disturbing its surface currents flow. Next, to optimize the performance in terms of isolation and compactness, several configurations of defected ground structures (DGS) are explored.

- Step 1–Connected ground plane (cross-shaped connection) [Fig 6}:

**Fig 6:**
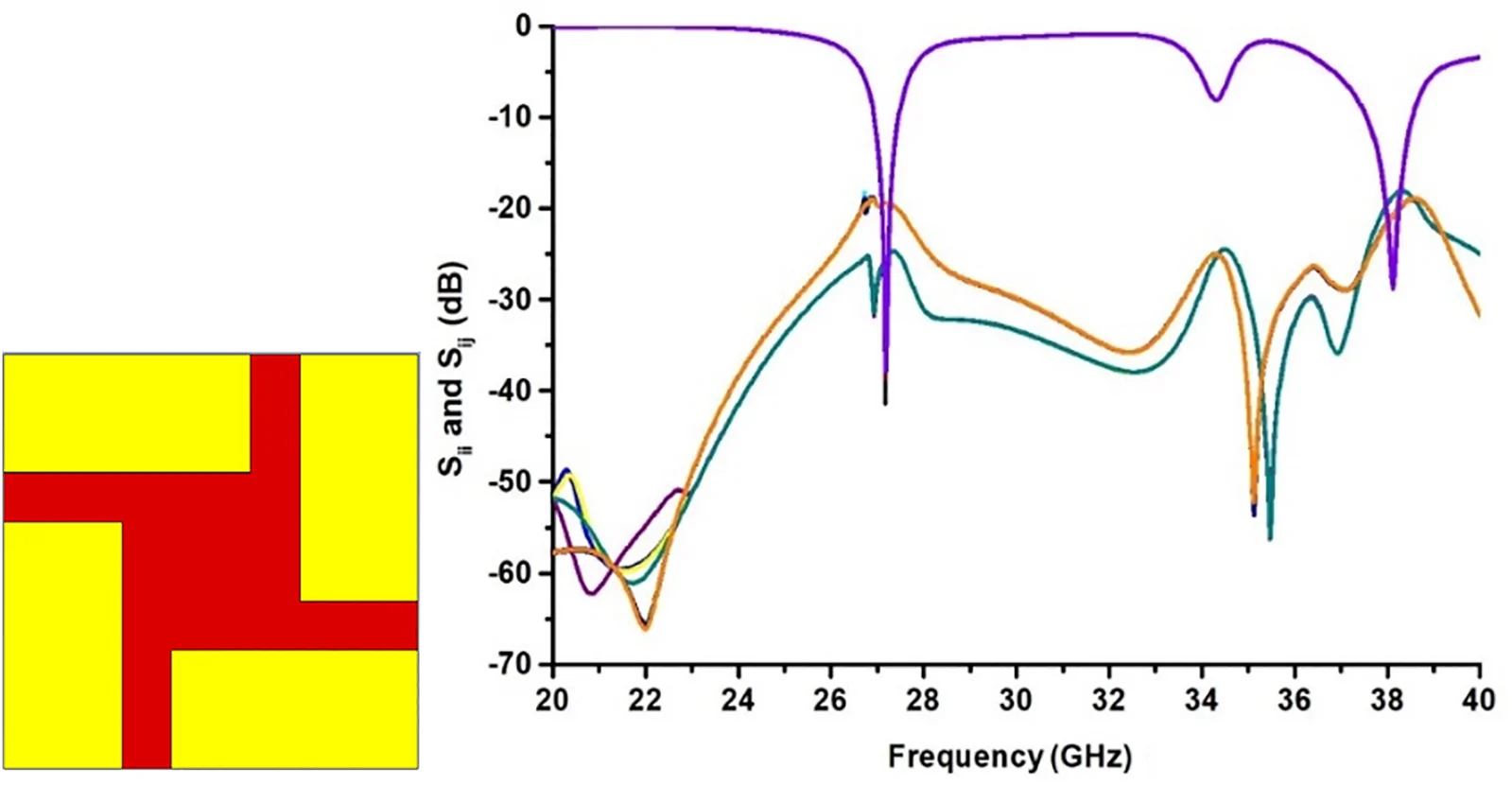
Analyzed results for connected ground plane configuration.

The modelling is initiated by connecting the four ground planes with a complete conductor base to form a continuous ground, as shown in Fig 6. Compared to the baseline of four isolated ground planes [Fig 7], the connected ground configuration reduces mutual coupling; this improvement is apparent when comparing the S-parameter plots for the connected and isolated cases [Fig 6 vs Fig 7].

**Fig 7:**
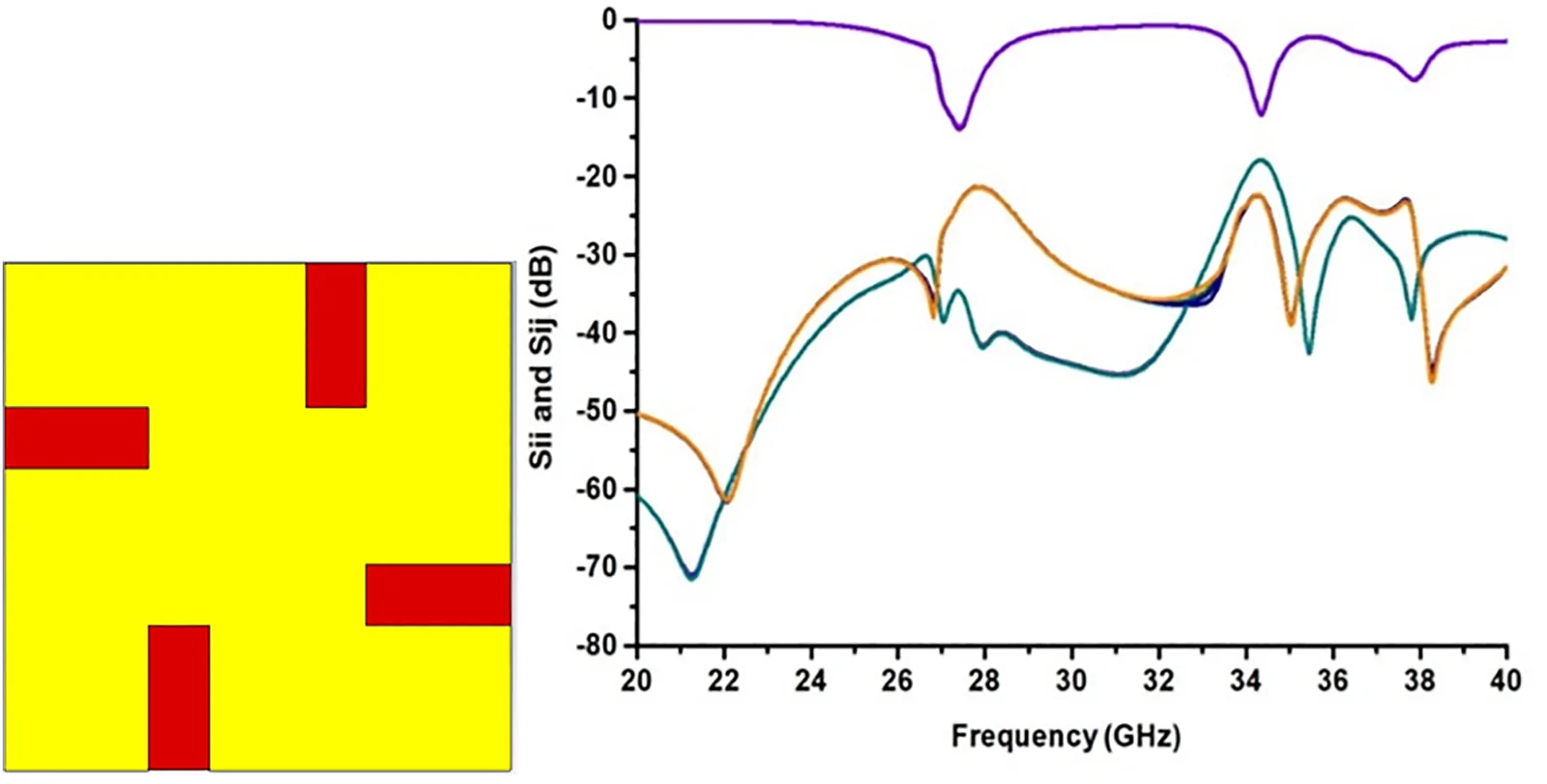
Analyzed results for isolated ground plane configuration.

- Step 2 – Circular-slot DGS [Fig 8]:

**Fig 8:**
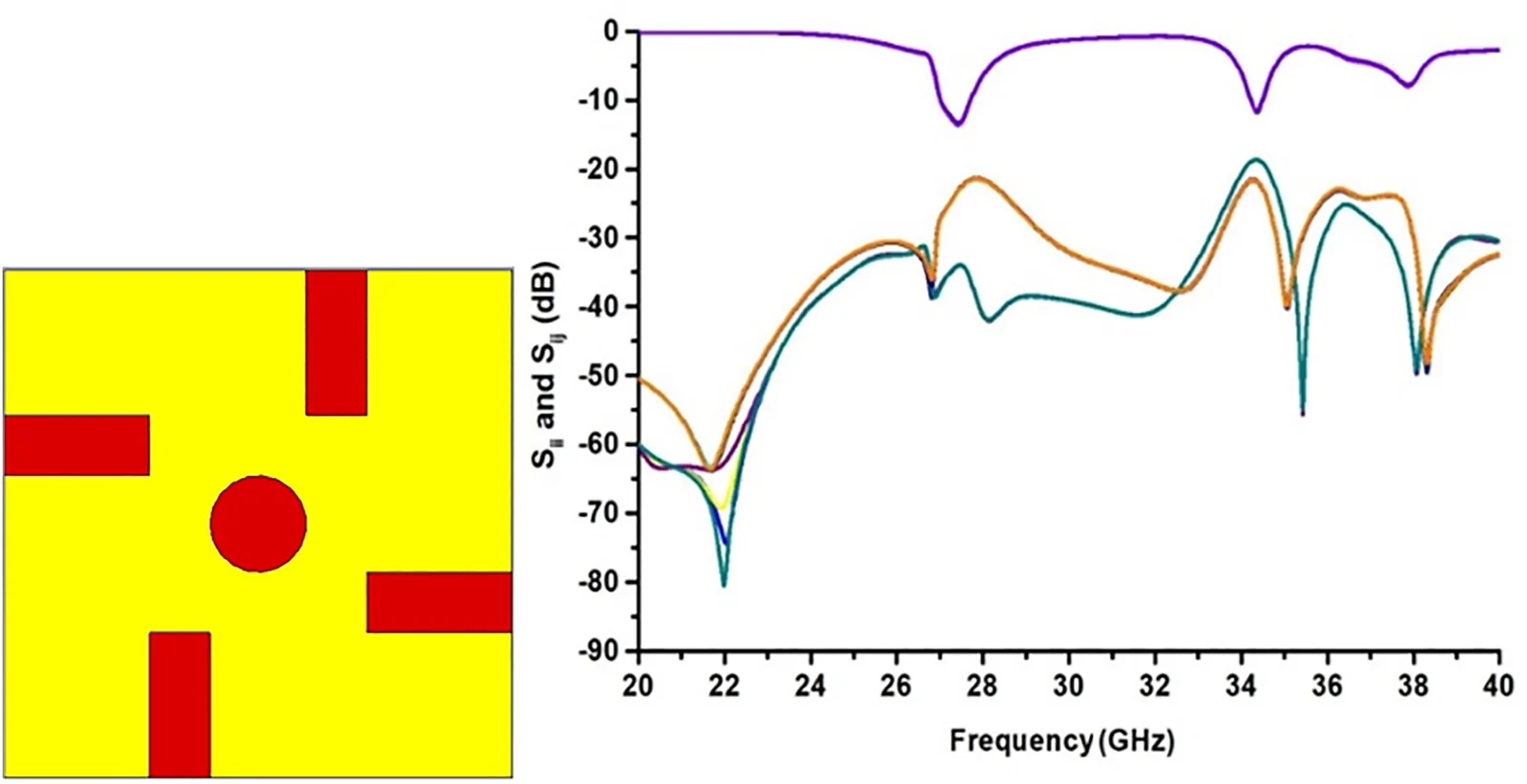
Analyzed results for circular slot DGS-based configuration.

A circular slot (radius R_DGS_ = 2 mm) is introduced into the baseline ground arrangement to form a circular-slot DGS [Fig 8]. The S-parameters for this case are shown in Fig 8. While the circular slot modifies the resonance locations and the impedance behaviour, it does not improve isolation relative to the connected ground configuration; in fact, the plots in Fig 8show increased coupling in some bands compared with Fig 6. This step is therefore important as it reveals the effect of a central circular perturbation on resonance tuning and coupling, even though it alone does not give the desired isolation improvement.

- Step 3–Trapezoidal-slot DGS [Fig 9

**Fig 9:**
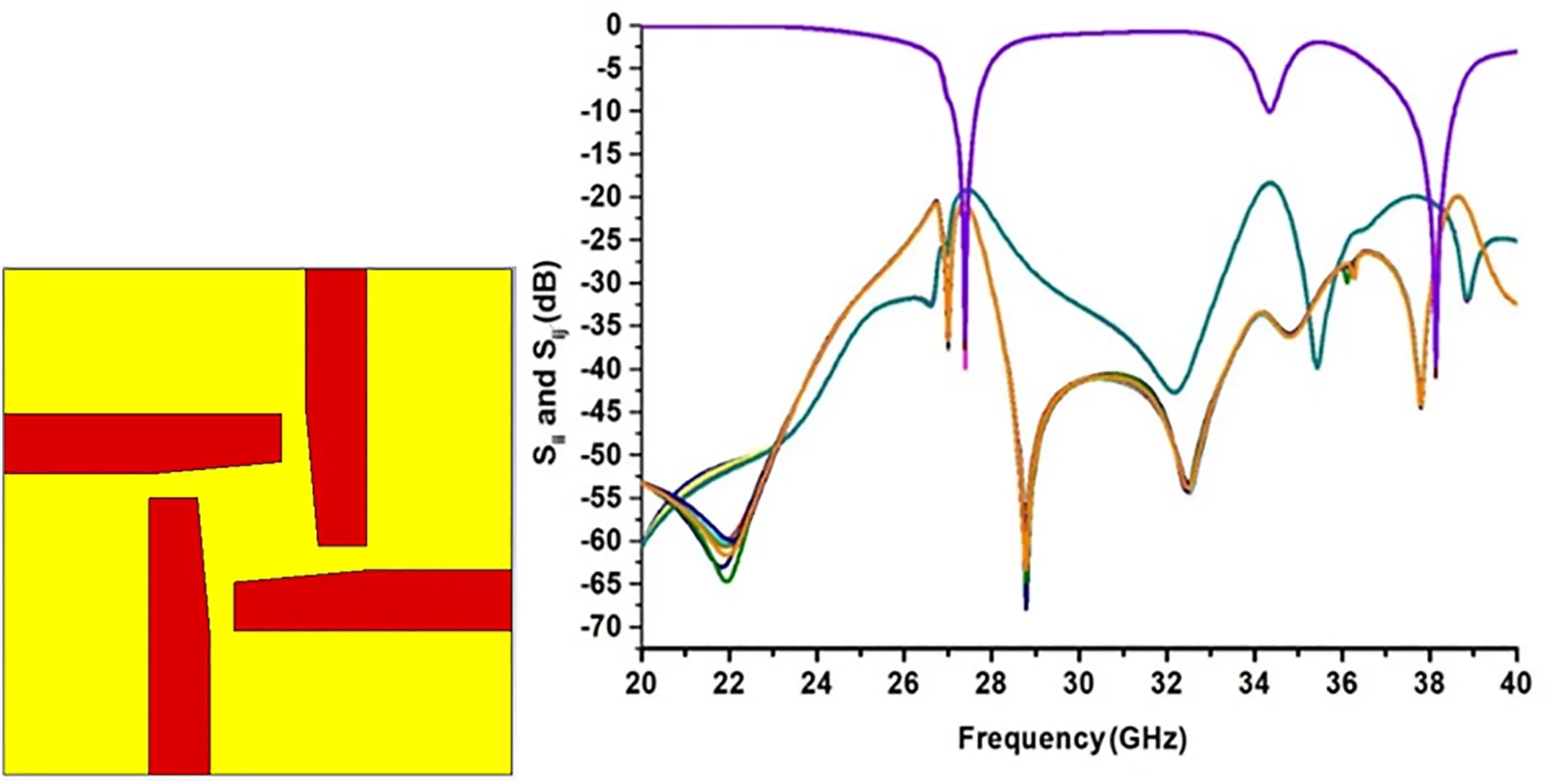
Analyzed results for Trapezoidal-slot DGS-based configuration.

Finally, four trapezoidal slots (dimensions h_Q_ = 2 mm, W_Q_ = 5.5 mm, H_Q_ = 2.5 mm) are placed symmetrically at each side of the baseline ground to form a trapezoidal-slot DGS. The S-parameters are presented in Fig 9. This modification is evaluated because it offers a different spectral perturbation than the circular slot; the results show how slot shape and placement influence both resonance alignment and inter-element coupling, guiding the choice of the final ground geometry.

- Final Step – Merged (hybrid) DGS Design [Fig 10]:

**Fig 10:**
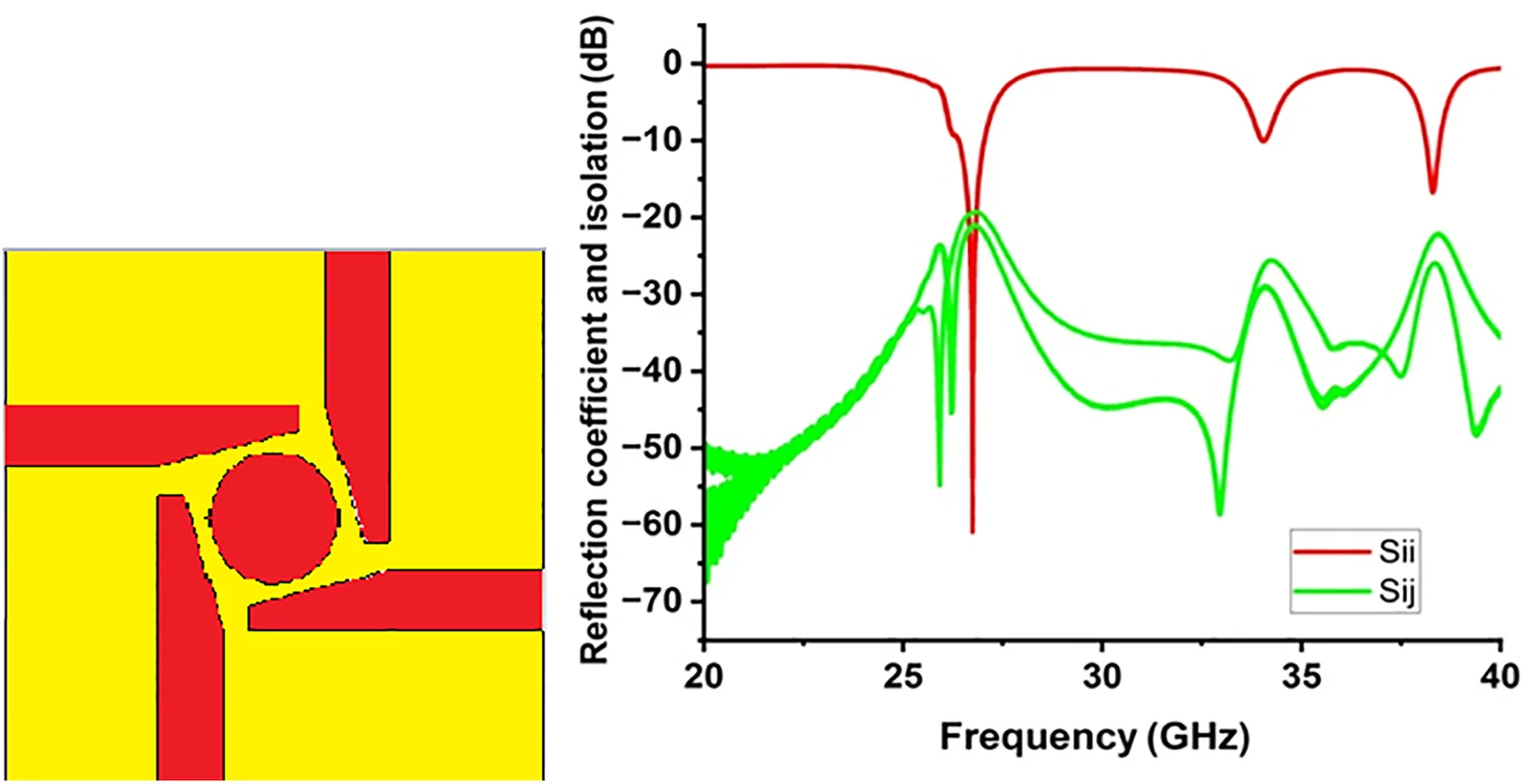
Analyzed results for proposed ground plane configuration.

Ultimately, the circular and trapezoidal slot designs are combined to form a hybrid DGS structure. This final configuration delivered the best performance, showing in Fig 10excellent isolation above 19.32 dB and strong dual-band operation at both 26.73 GHz and 38.29 GHz.

This systematic exploration confirmed that while some DGS configurations degraded performance, the optimized merged design achieved significant improvements in bandwidth, isolation, and overall MIMO efficiency.

### 4.3. Effect of radius of DGS circle (R_DGS_)

When the radius of the DGS circle (R_DGS_) changes from 1.6 mm to 2.3 mm, Fig 11 and Table 3 shows that the lower resonant frequency is affected significantly, with little impact on the higher band resonance frequency. However, prominent variations in both the reflection and isolation parameters are clearly noted due to changes in R_DGS_. The best reflection coefficient and hence best impedance matching is obtained for the proposed dimension of R_DGS_ = 2 mm, dropping to −60.94 dB at 26.73 GHz and −16.80 dB at 38.29 GHz, respectively. The operating bandwidths are 0.65 GHz (26.39–27.04 GHz) and 0.36 GHz (38.11–38.47 GHz) at 26.73/38.29 GHz, respectively. This indicates that the radius of circular-shaped DGS, R_DGS_ = 2 mm, is utilized in the proposed design. In addition, the suggested design with R_DGS_ = 2 mm ensures an isolation of greater than 19.32 dB among the various radiating elements. At 26.73 GHz, a gain of 4.80 dBi and 97.61% radiation efficiency, and at 38.29 GHz, 7.61 dBi gain and 95.45% radiation efficiency are recorded. The proposed R_DGS_ produces excellent results and improves isolation, as compared to other tested dimensions.

**Table 3:** Simulation results with different R_DGS_ dimensions.

R_DGS_ (mm)	1.6		2 (proposed)		2.3	
Frequency (GHz)	27.43	38.23	26.73	38.29	27.47	38.23
Bandwidth (GHz)–	0.48	0.98	0.65	0.36	0.48	0.98
–10 dB IBW (GHz)	27.21-27.69	37.65-38.63	26.39-27.04	38.11-38.47	27.21-27.69	37.63-38.61
Gain (dBi)	4.32	6.53	4.80	7.61	4.21	6.32
Sii (dB)	–34.87	–29.49	–60.94	–16.80	–27.86	–30.60
Isolation (dB)	>17.5	>20.42	>19.32	>22.12	>20.48	>21.7
Rad. Efficiency (%)	95%	94.1%	97.61%	95.45%	95.21%	93.90%

**Fig 11:**
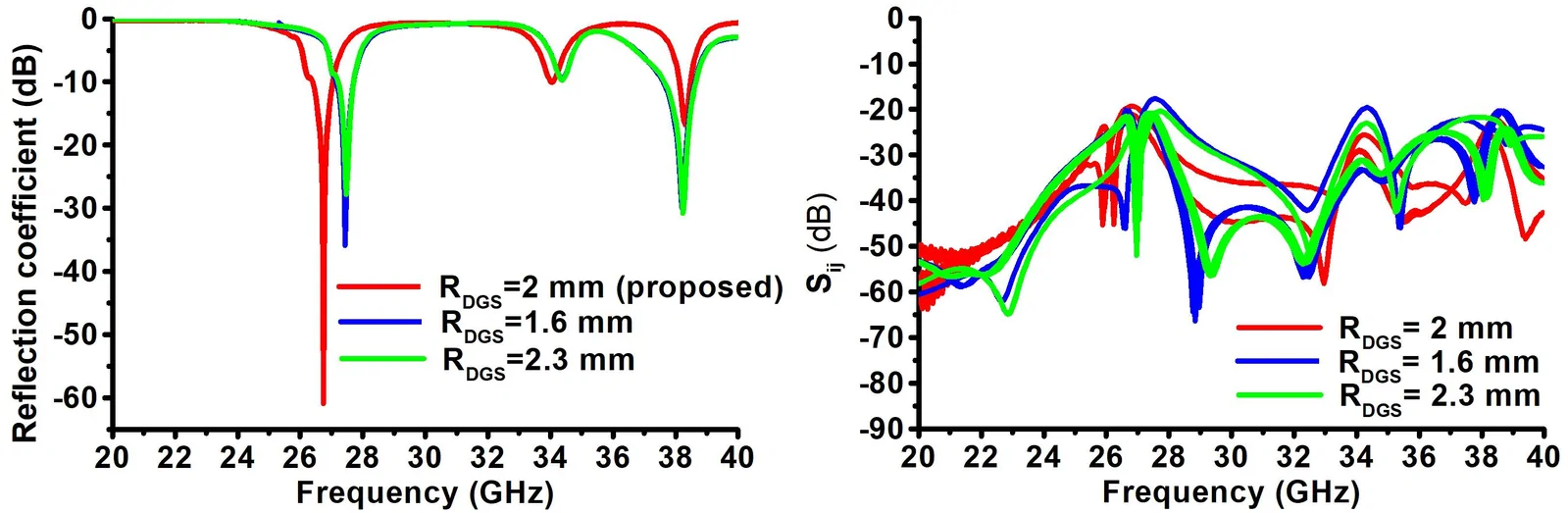
Effects of variations in R_DGS_: reflection coefficients and isolations.

### 4.4. Effect of the short base (h_Q_) of DGS trapezoidal

There is a noticeable downward shift in the lower resonant frequency along with significant improvement in reflection coefficient due to an increment (from 1 mm to 2 mm) in the dimensions of the short base (h_Q_) of the DGS. Similarly, the isolation among patch elements varies due to changes in h_Q_ parameter. According to Fig 12 and Table 4, the reflection coefficient drops to −60.94 dB at 26.73 GHz and −16.80 dB at 38.29 GHz, respectively, when the short base (h_Q_) of the DGS trapezoidal is proposed to be 2 mm, and the impedance bandwidth is around 0.65 GHz (26.39–27.04 GHz) and 0.36 GHz (38.11–38.47 GHz) at 26.73/38.29 GHz, respectively. It exhibits a gain of 4.80/7.61 dBi at 26.73/38.29 GHz, respectively. The corresponding radiation efficiencies are 97.61 and 95.45%, respectively at 26.73 and 38.29 GHz. Although the proposed radiator with other values of h_Q_ provides improved isolation, the proposed h_Q_ of 2 mm yields the optimal balance of impedance matching, gain, radiation efficiency, and isolation.

**Table 4:** Simulated results utilizing various h_Q_ Dimensions.

h_Q_ (mm)	1		1.5		2 (proposed)	
Frequency (GHz)	27.51	38.1	27.51	38.3	26.73	38.29
Bandwidth (GHz)	0.50	0.88	0.50	0.90	0.65	0.36
−10 dB IBW (GHz)	27.25-27.75	37.65-38.53	27.24-27.74	37.66-38.56	26.39-27.04	38.11-38.47
Gain (dBi)	4.53	6.22	4.57	6.56	4.80	7.61
Sii (dB)	− 30.60	−25.66	−30.59	−35.51	−60.94	−16.80
Isolation (dB)	>20.3	>20.1	>19.5	>23	>19.32	>22.12
Rad. Efficiency (%)	95.5%	93.6%	95.15%	93.55%	97.61%	95.45%

**Fig 12:**
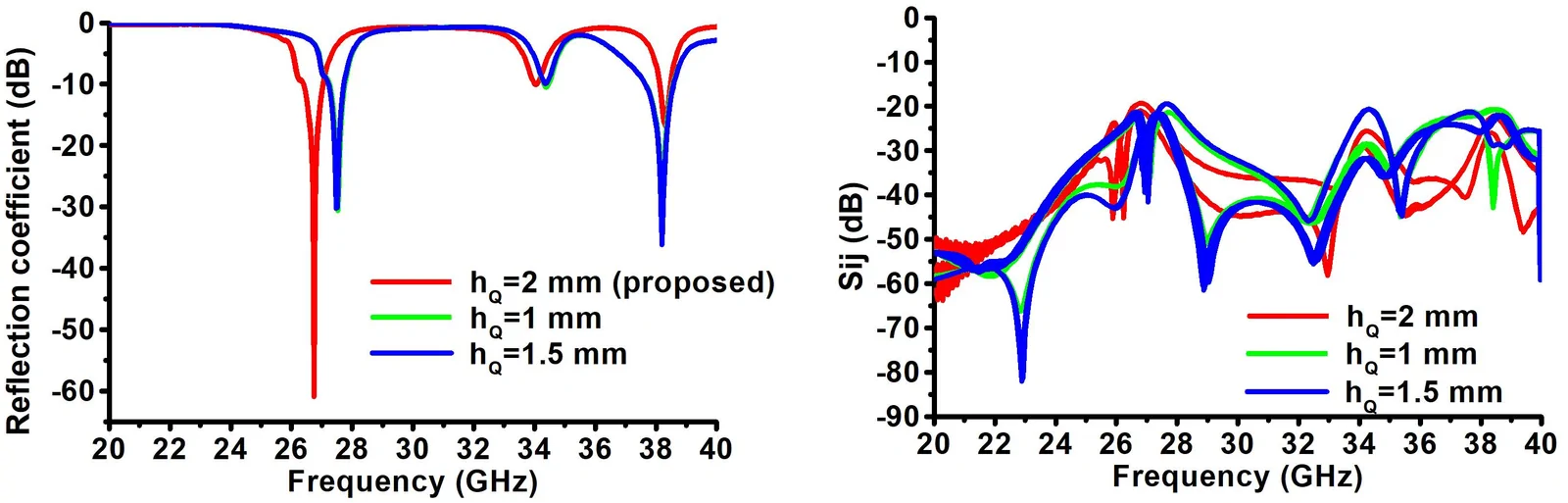
Simulation analysis of h_Q_: changes in reflection coefficient and isolation.

### 4.5. Effect of the horizontal base (W_Q_) of the DGS trapezoidal

The resonant response undergoes a notable change in operational bandwidth and impedance matching when the W_Q_ is changed from W_Q_ = 4.5 mm to W_Q_ = 6 mm. Although the designed antenna shows improved isolation for W_Q_ = 4.5 mm, other crucial parameters like reflection coefficient, gain, radiation efficiency, operating bandwidth deteriorate along with frequency shifts for this case. Further increment of W_Q_ to 6 mm also leads to unsatisfactory results, as summarized in Table 5. When the proposed horizontal base (W_Q_) of the DGS trapezoidal is 5.5 mm, the reflection coefficient improves to −60.94 and −16.80 dB, respectively at 26.73 and 38.29 GHz [Fig 13 and Table 5]. At 26.73 GHz, the impedance bandwidth is around 0.65 GHz (26.39 to 27.04 GHz), and at 38.29 GHz, it is about 0.36 GHz (38.11 to 38.47 GHz). The analyzed results show a gain of 4.80 dBi along with 97.61% radiation efficiency at 26.73 GHz and at 38.29 GHz, these values correspond to 7.61 dBi and 95.45%, respectively. Hence, the antenna with proposed W_Q_ of 5.5 mm provides an overall balanced performance in comparison to the other two values of W_Q_.

**Table 5:** Simulated Results by varying the values of W_Q._

W_Q_ (mm)	4.5		5.5 (proposed)		6	
Frequency (GHz)	27.37	37.77	26.73	38.29	27.39	38.21
Bandwidth (GHz)	0.70	0.82	0.65	0.36	0.42	0.74
−10 dB IBW (GHz)	26.97-27.67	37.37-38.19	26.39-27.04	38.11-38.47	27.17-27.59	37.77-38.51
Gain (dBi)	4.03	5.55	4.80	7.61	4.33	7.14
Sii (dB)	−12.1	−26.80	−60.94	−16.80	−18.96	−22.12
Isolation (dB)	>20.07	>21.10	>19.32	>22.12	>19.41	>17.38
Rad. Efficiency (%)	95.86%	94.66%	97.61%	95.45%	96.43%	95.22%

**Fig 13:**
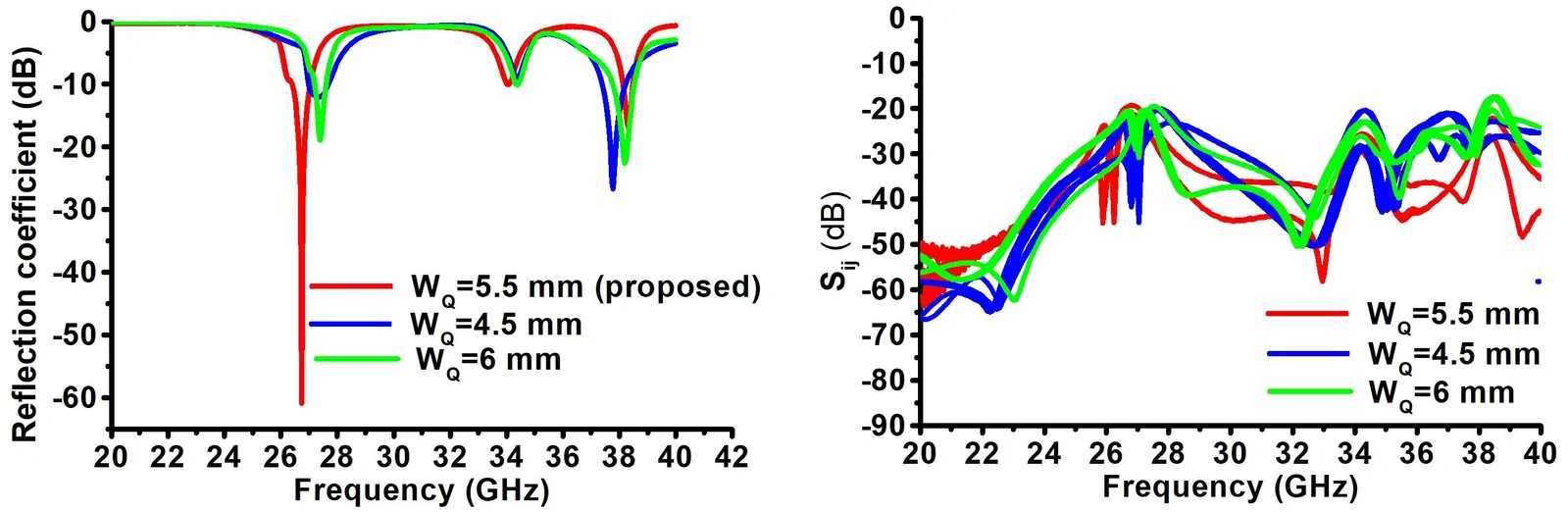
Simulation results due to change in W_Q_: Reflection coefficient and Isolation.

## 5. Measurement results and discussion

The prototype of the designed 4-port MIMO antenna system is fabricated through standard chemical etching process for its experimental validation purpose, as demonstrated in Fig 14. It was experimentally tested using a vector network analyzer, while simulations were conducted using CST Microwave Studio. Fig 15 provides a comparison between simulated and measured reflection (S_11_). Figs 16–21 present the transmission (S_21_, S_31_, etc.) coefficients of the proposed antenna. The measured S_11_ shows good agreement with the simulated results, with both exhibiting resonance at 26.73 GHz and 38.29 GHz, confirming dual-band operation. Small discrepancies between the computed and tested values can be explained by the tolerances in fabrication and experimental process. Nevertheless, the reflection coefficients (S_ii_) remain below −10 dB across the intended dual frequency bands, and isolation between ports stays higher than −19 dB, indicating the effectiveness of the design and its suitability for dual band 5G mmWave applications.

**Fig 14:**
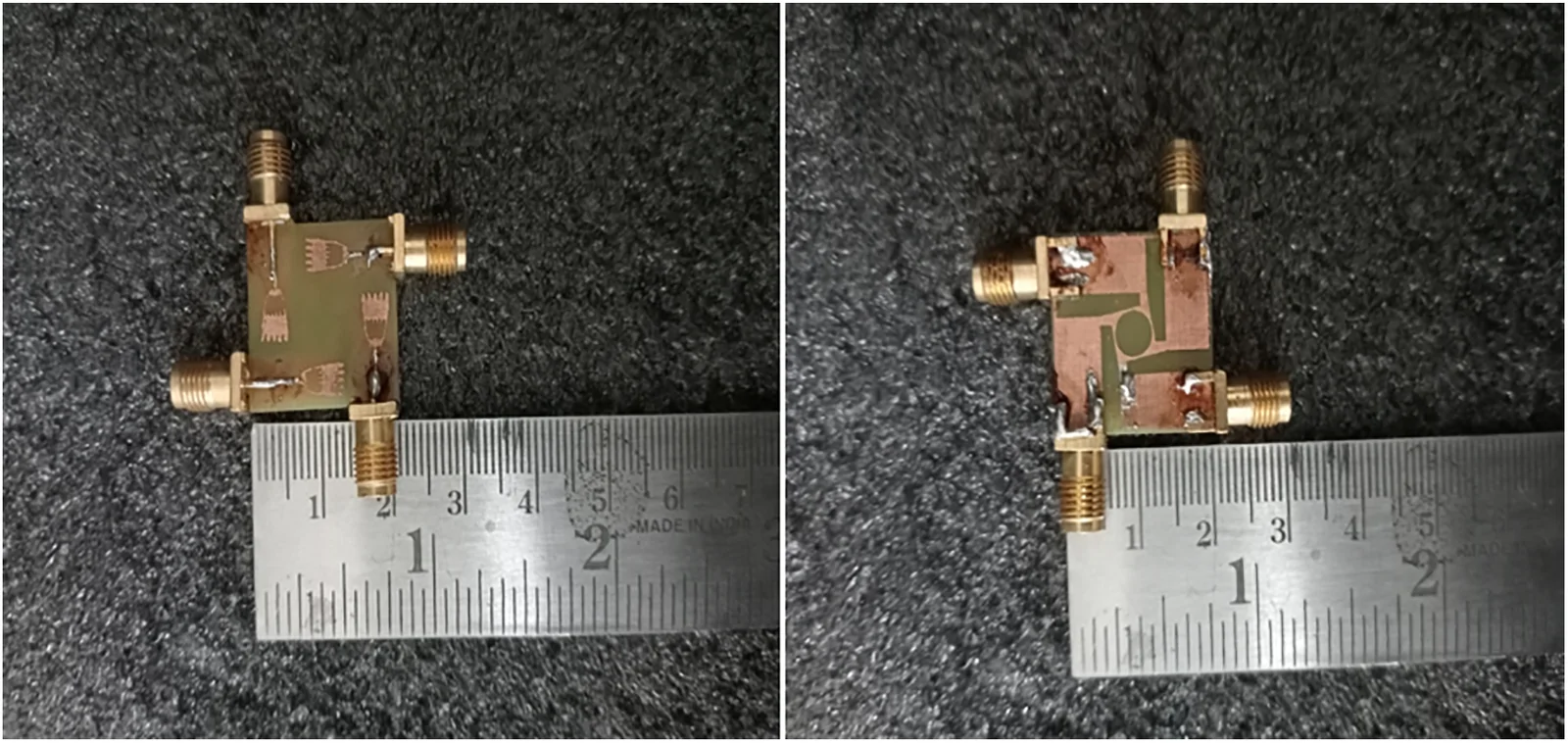
Images of the fabricated prototype: Patch elements and ground plane.

**Fig 15:**
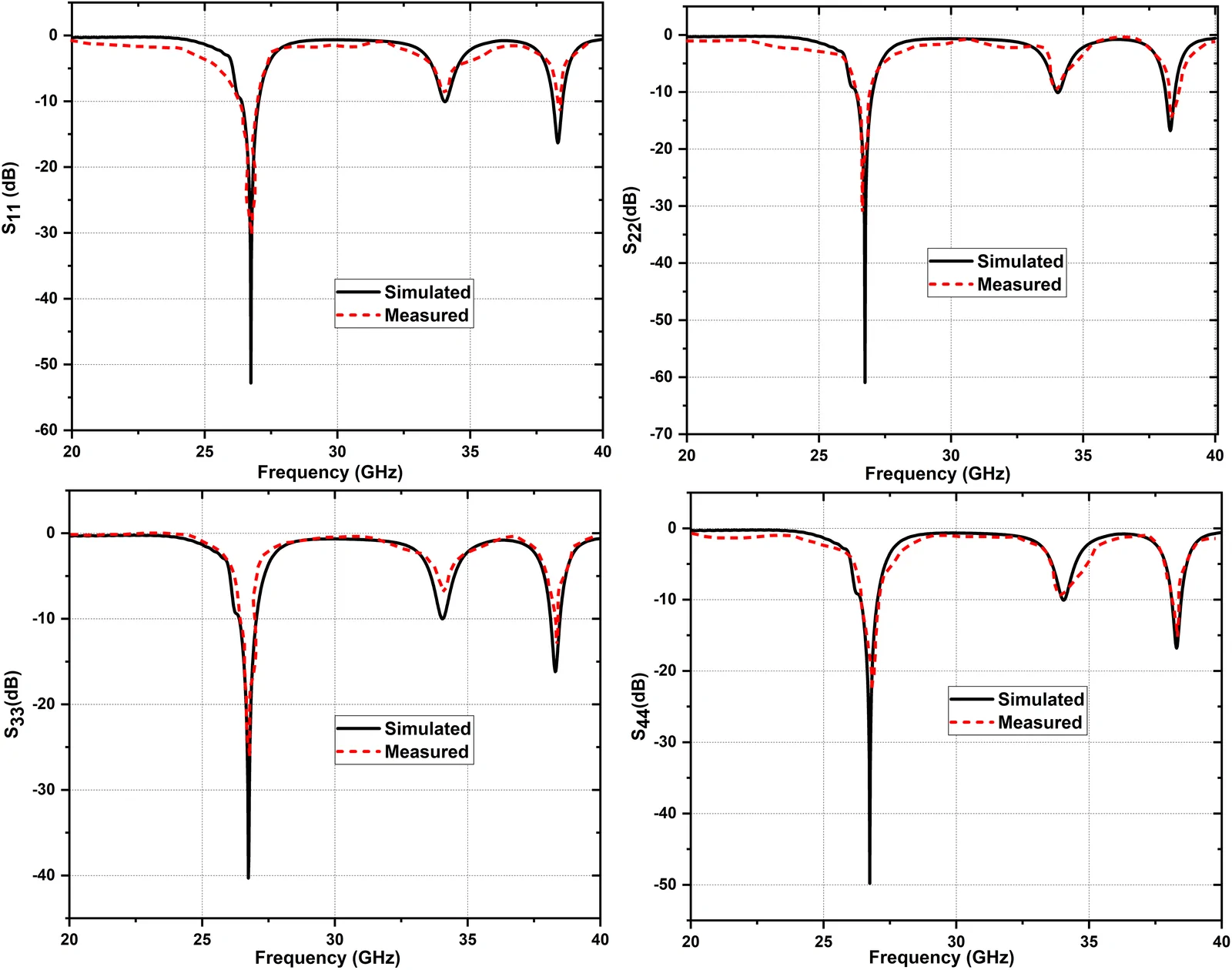
Comparison of reflection coefficients for the suggested four-port MIMO antenna.

**Fig 16:**
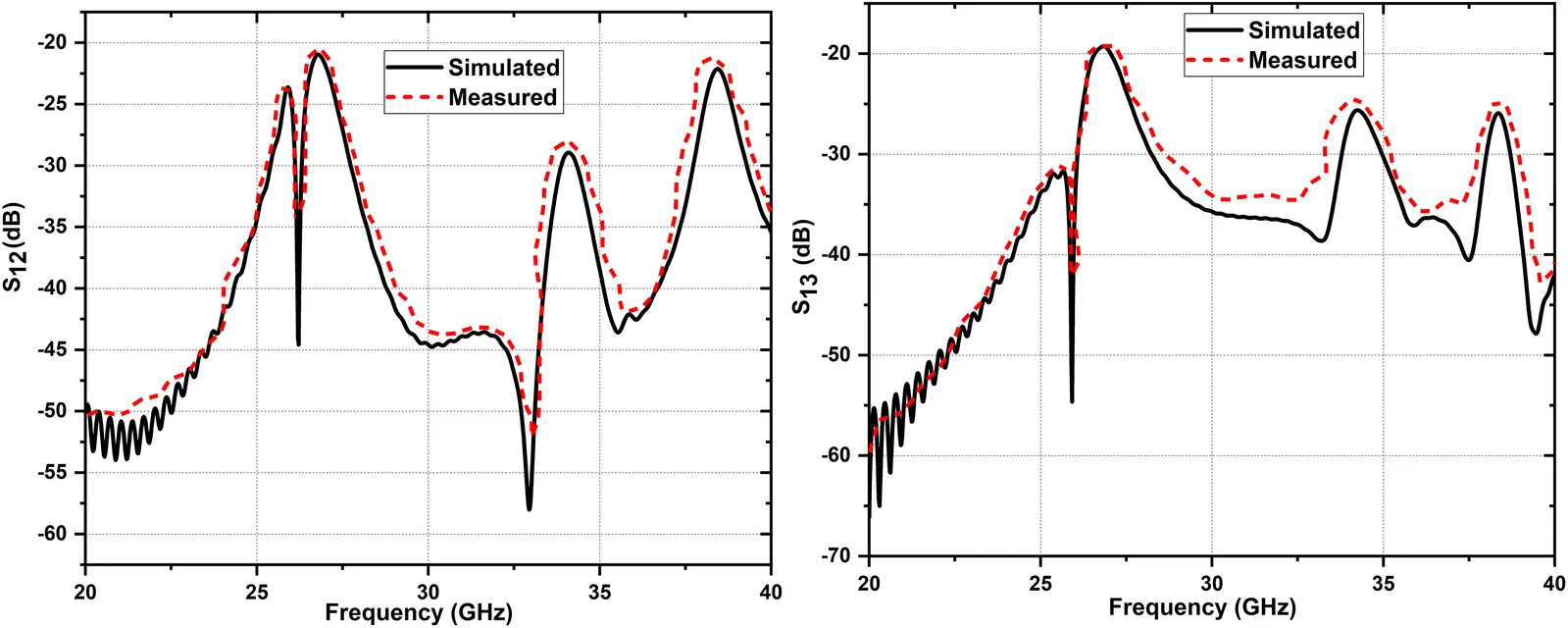
Comparison of measured and simulated S_12_ and S_13_.

**Fig 17:**
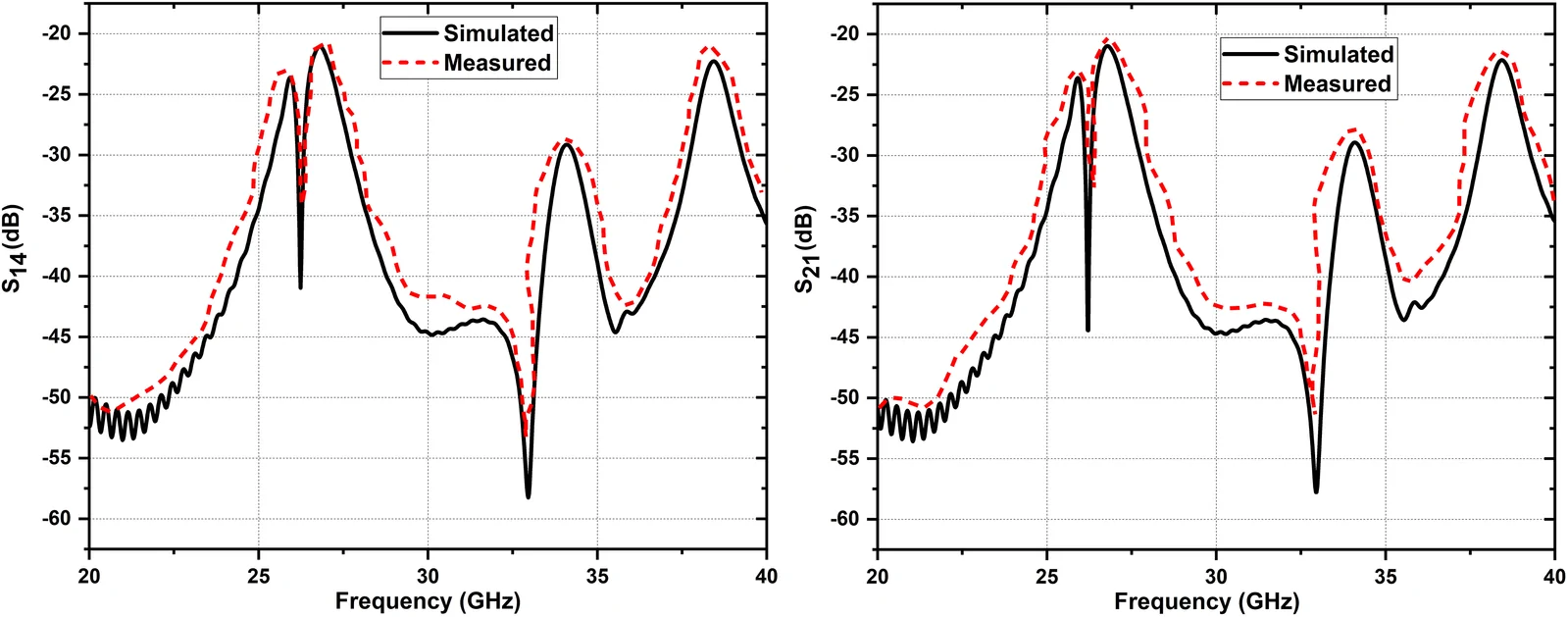
Comparison of measured and simulated S_14_ and S_21_.

**Fig 18:**
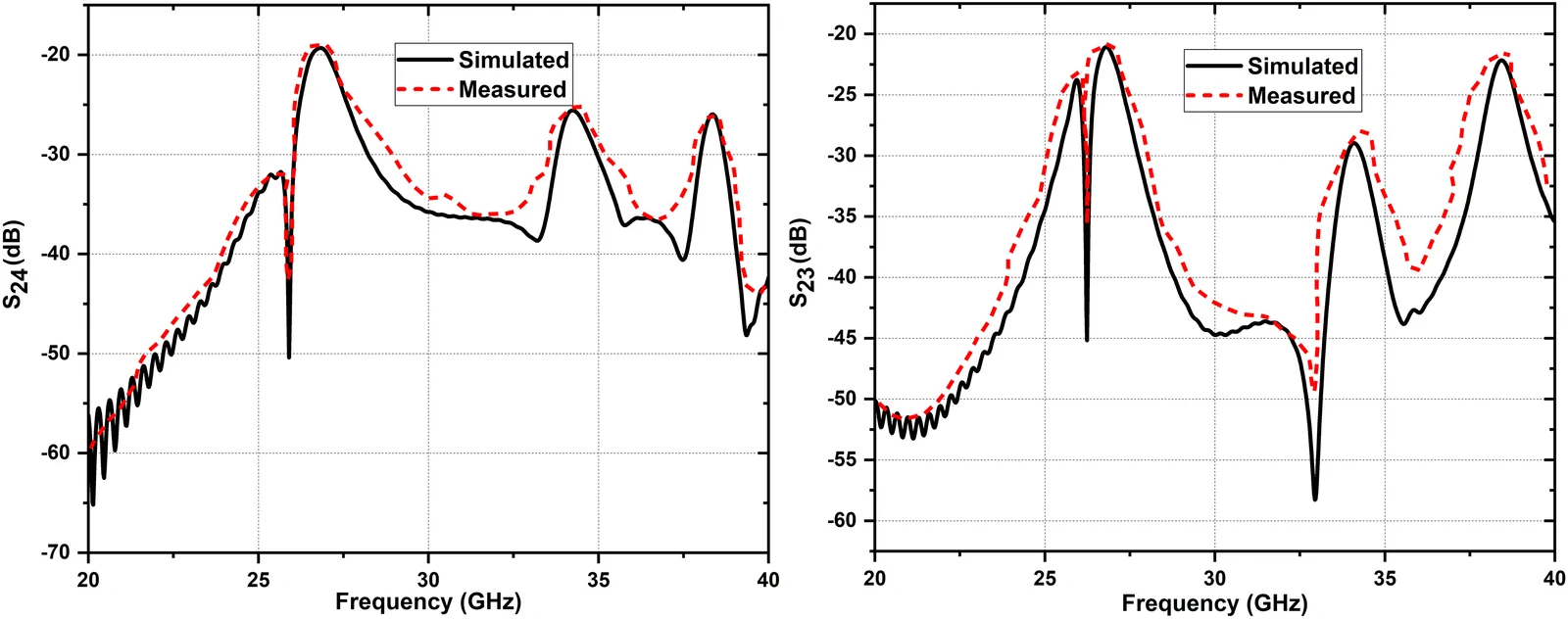
Comparison of measured and simulated S_24_ and S_23_.

**Fig 19:**
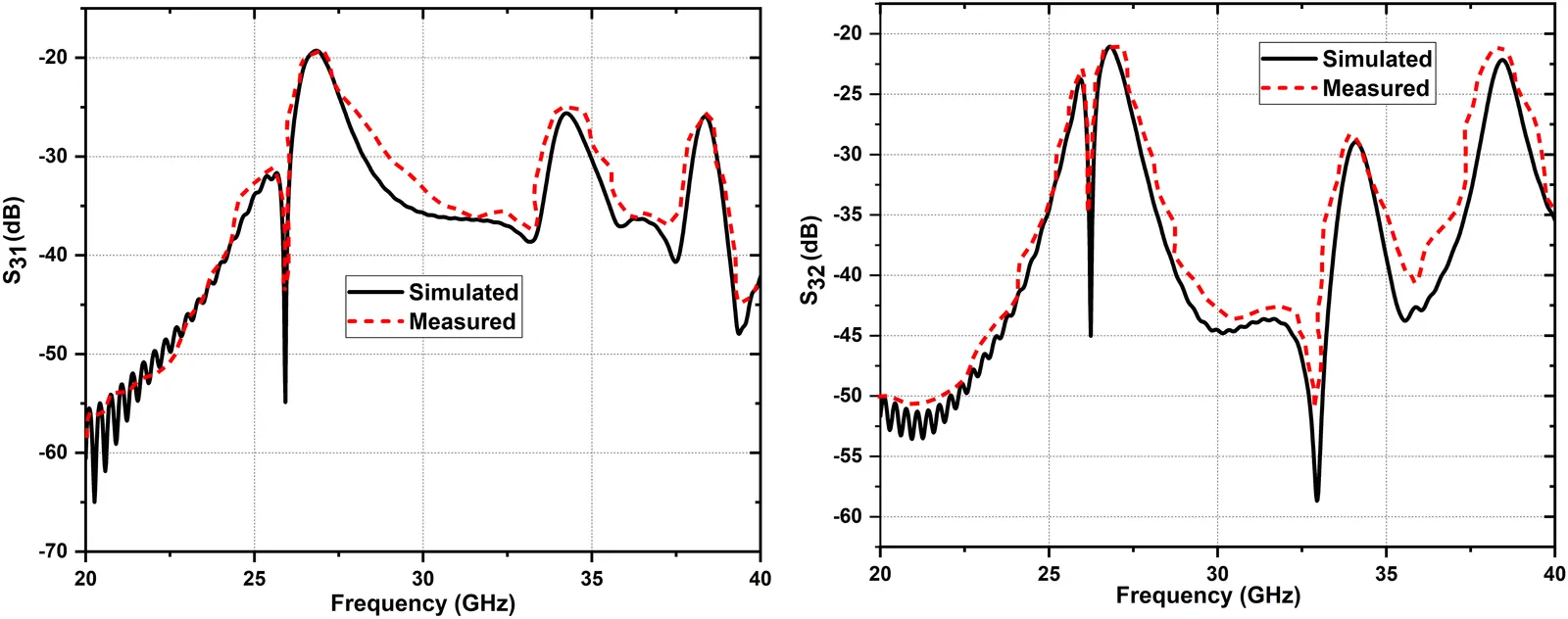
Comparison of measured and simulated S_31_ and S_32_.

**Fig 20:**
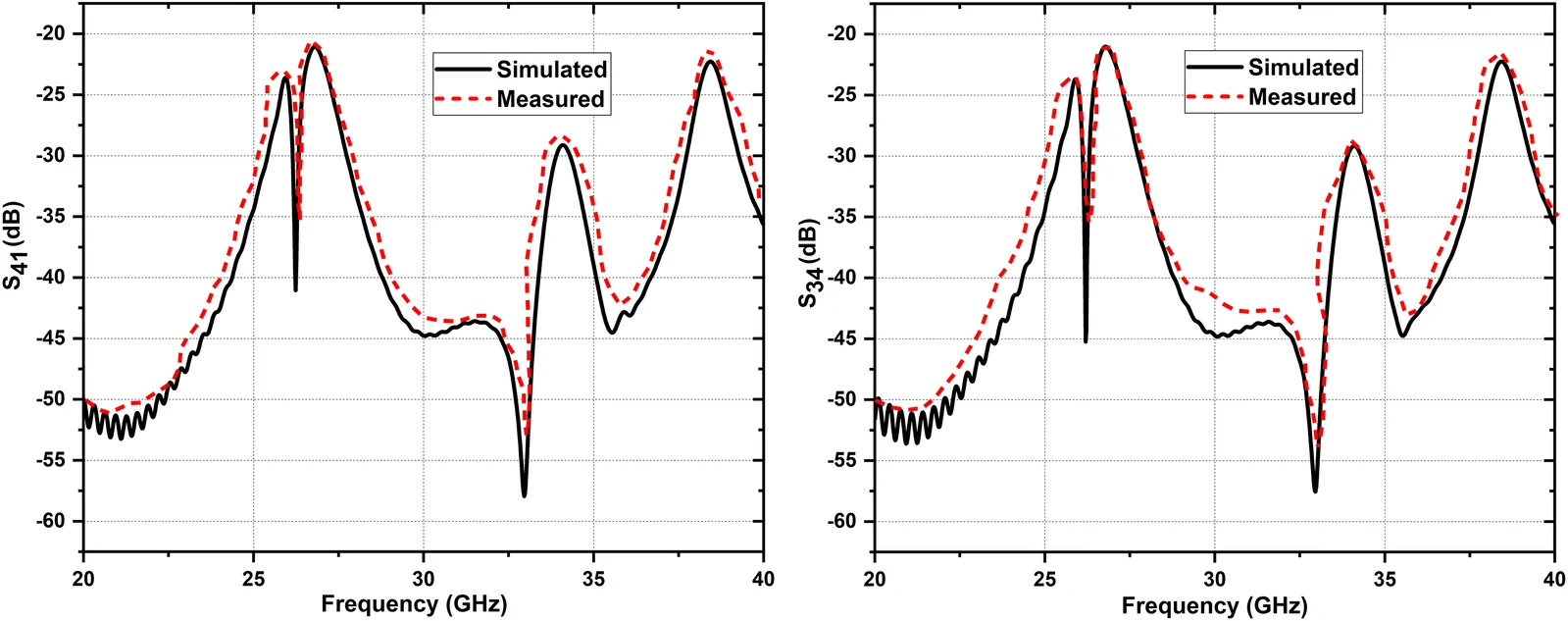
Comparison of measured and simulated S_41_ and S_34_.

**Fig 21:**
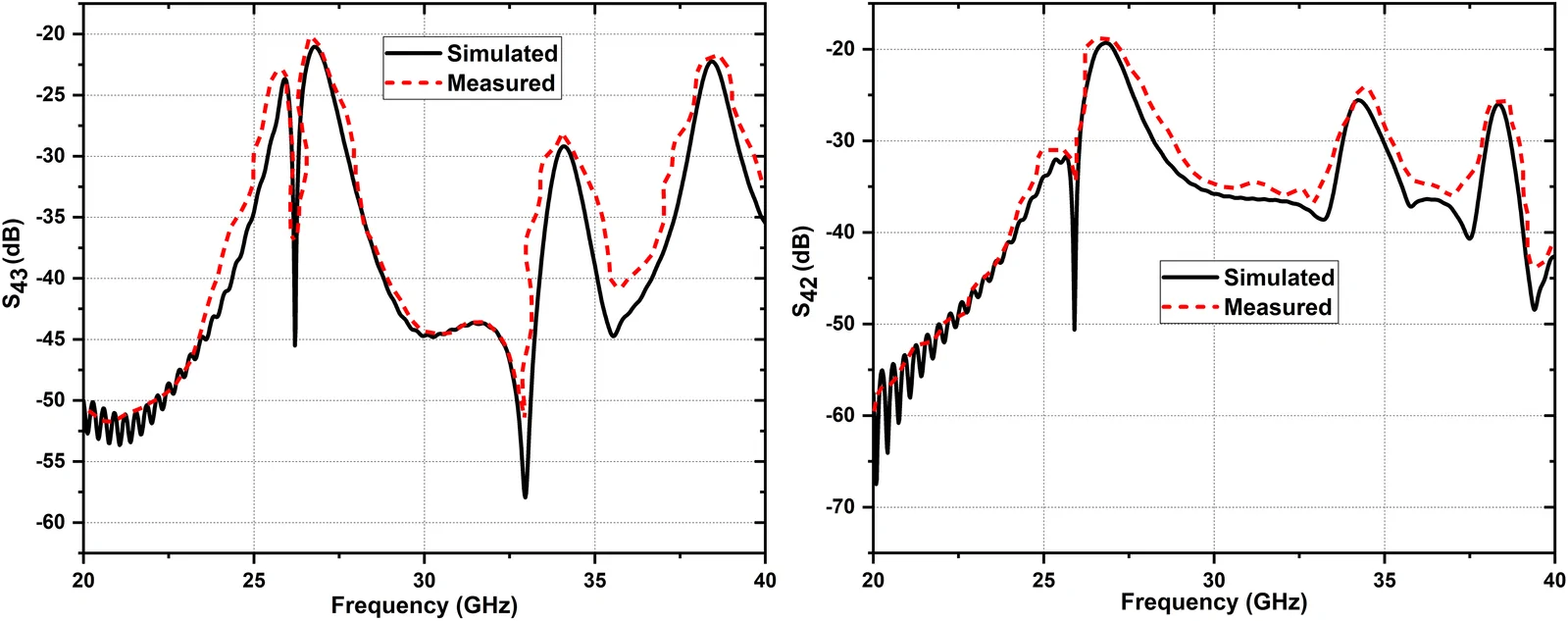
Comparison of measured and simulated S_43_ and S_42_.

Fig 22 highlights the gain and radiation efficiency plots for the presented 5G MIMO antenna, comparing both the simulation and experimental results. The simulated gains are 4.80/7.61 dBi, respectively at 26.73/38.29 GHz, with corresponding radiation efficiencies of 97.61% and 95.45%. The measured gains are respectively 4.63/7.22 dBi at 26.73/38.29 GHz, while the measured radiation efficiency is 94.12% at 26.73 GHz and 95.21% at 38.29 GHz. The simulated and measured results reported in Fig 22 show a strong agreement in terms of overall trends and antenna performance behavior. Although some numerical differences exist between simulation and measurement, these variations are expected due to practical implementation effects. The simulated results represent an ideal electromagnetic model, while the measured results corresponding to the fabricated prototype are possibly affected by fabrication tolerances, substrate’s dielectric-property variations, copper roughness, etching inaccuracies, SMA connector soldering effects, cable and connector losses, antenna alignment errors, residual reflections in the anechoic chamber. However, the experimental outcomes remain in acceptable agreement with the simulations and confirm the dual band operating mechanism and the effectiveness of the presented MIMO antenna model. The radiation patterns are measured in an anechoic chamber with co and cross pol components. Figs 23–26 present both co and cross pol E and H plane patterns at 26.73/38.29 GHz, showing consistent outcomes. The radiation patterns on the E and H planes covers the radiated regions with almost omnidirectional shapes with the formation of lobes.

**Fig 22:**
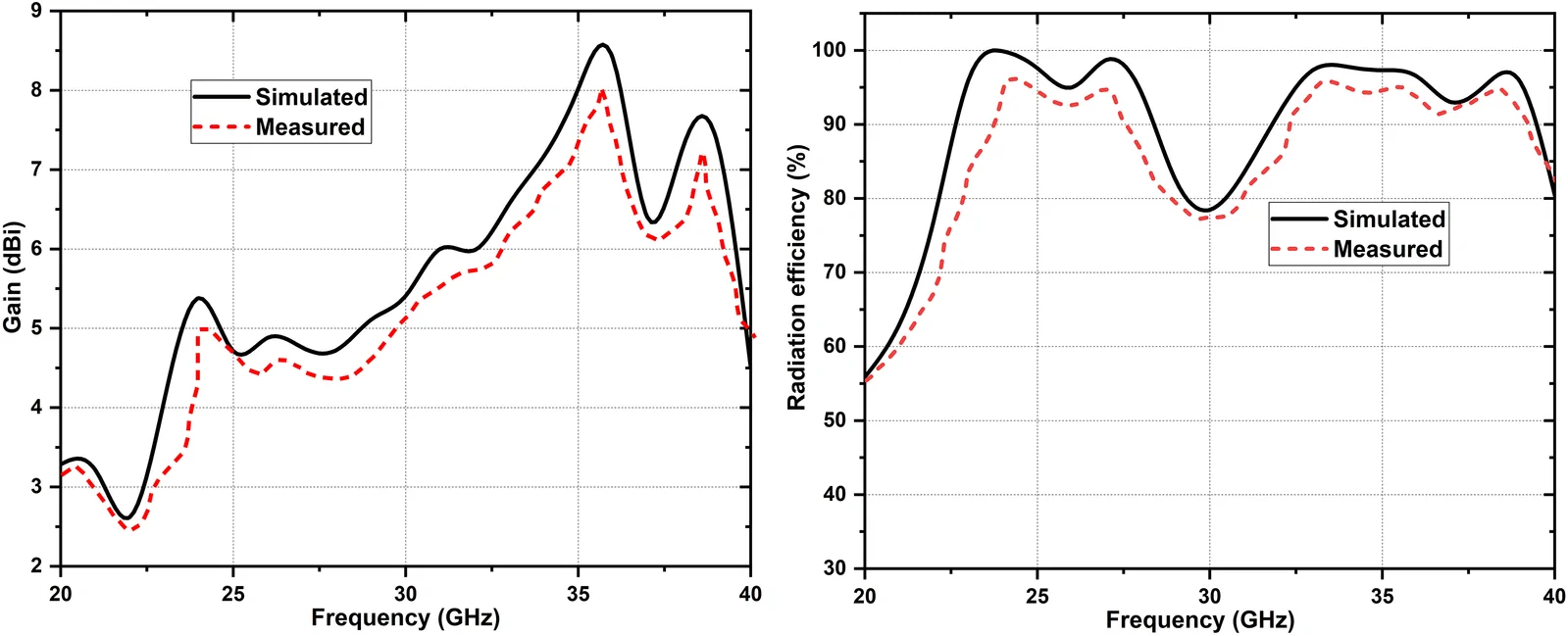
Comparison of radiation parameters of the presented antenna: Gain (dBi) and Radiation efficiency (%).

**Fig 23:**
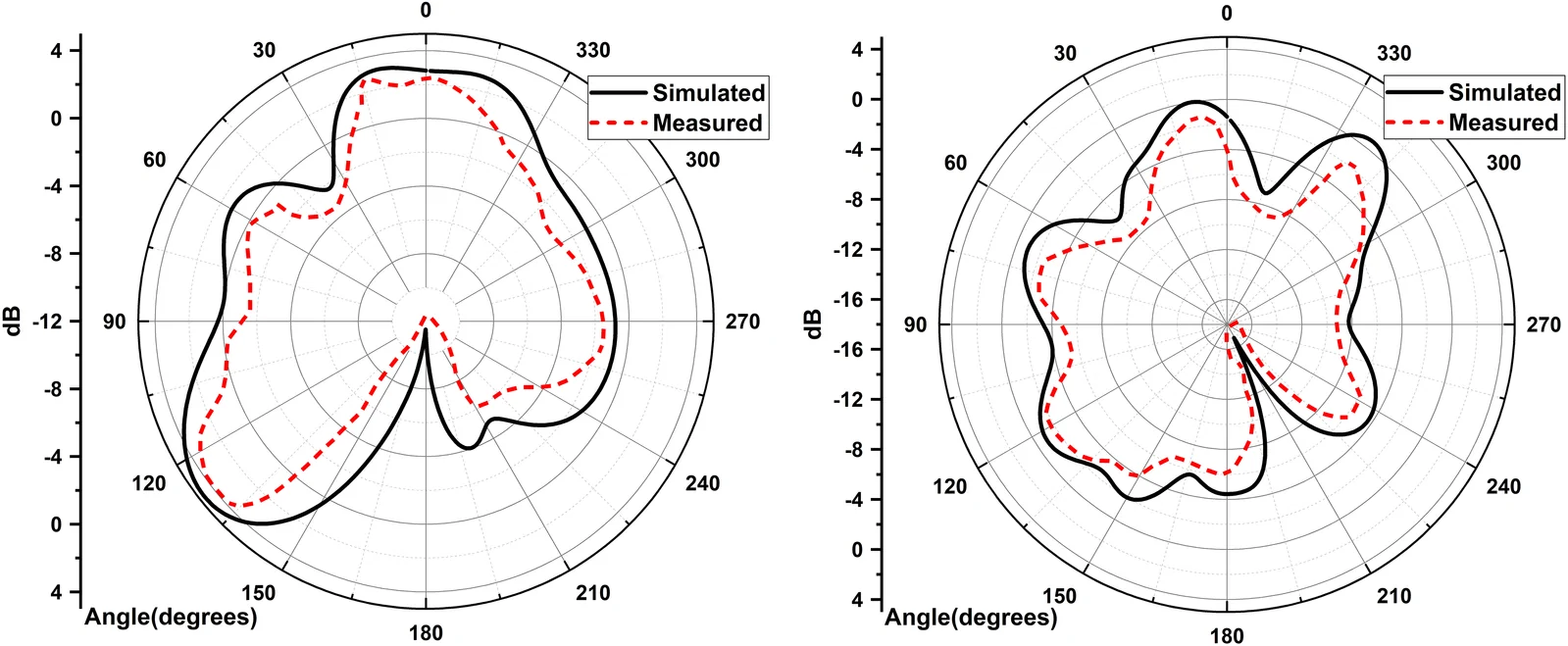
The radiation patterns (measured and simulated): Co pol phi at 26.73 GHz and Co pol phi at 38.29 GHz.

**Fig 24:**
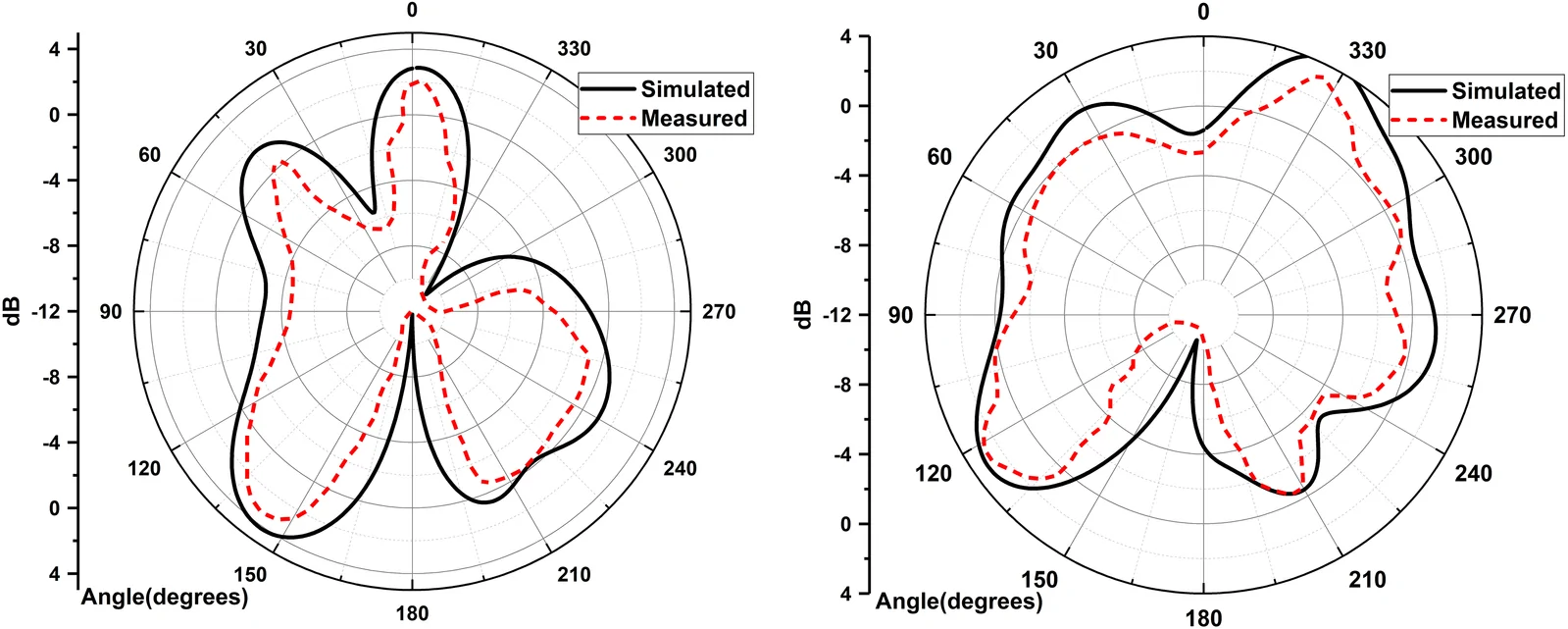
The radiation patterns (measured and simulated): Co pol Theta at 26.73 GHz and Co pol Theta at 38.29 GHz.

**Fig 25:**
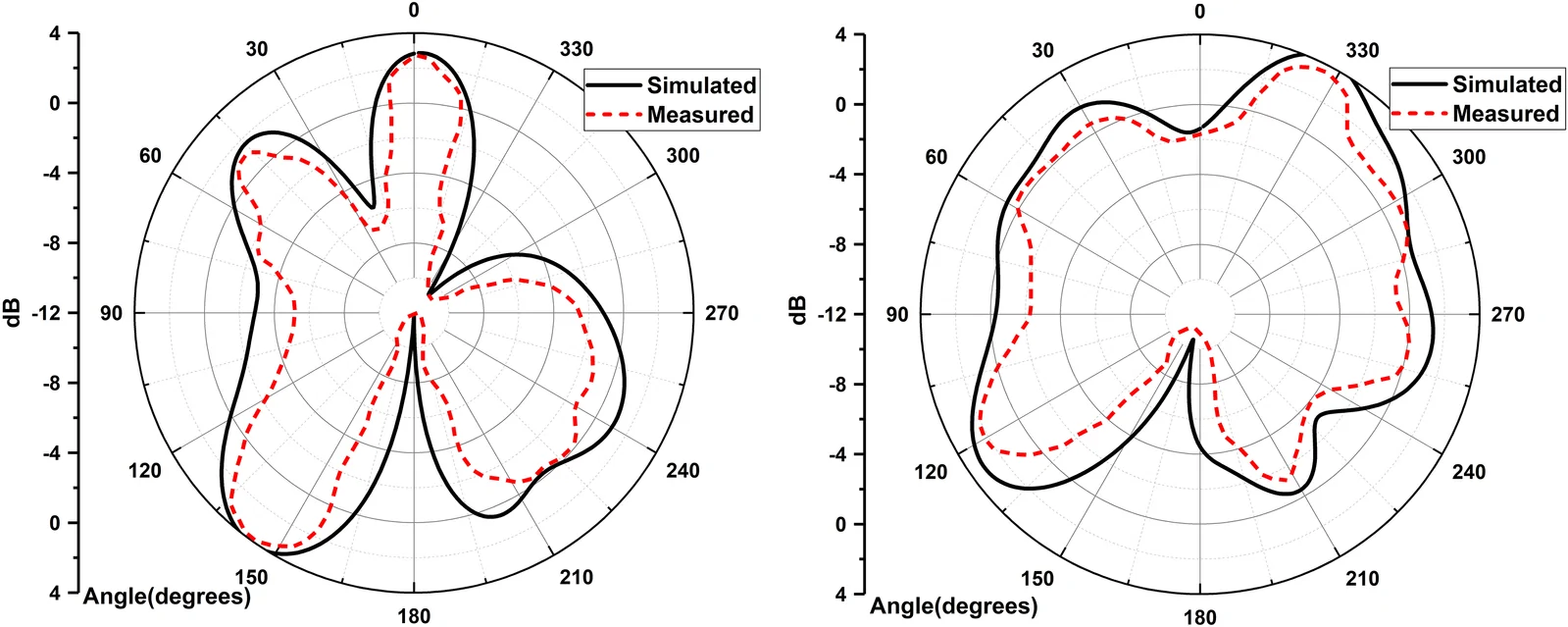
The radiation patterns (measured and simulated): Cross pol phi at 26.73 GHz and Cross pol phi at 38.29 GHz.

**Fig 26:**
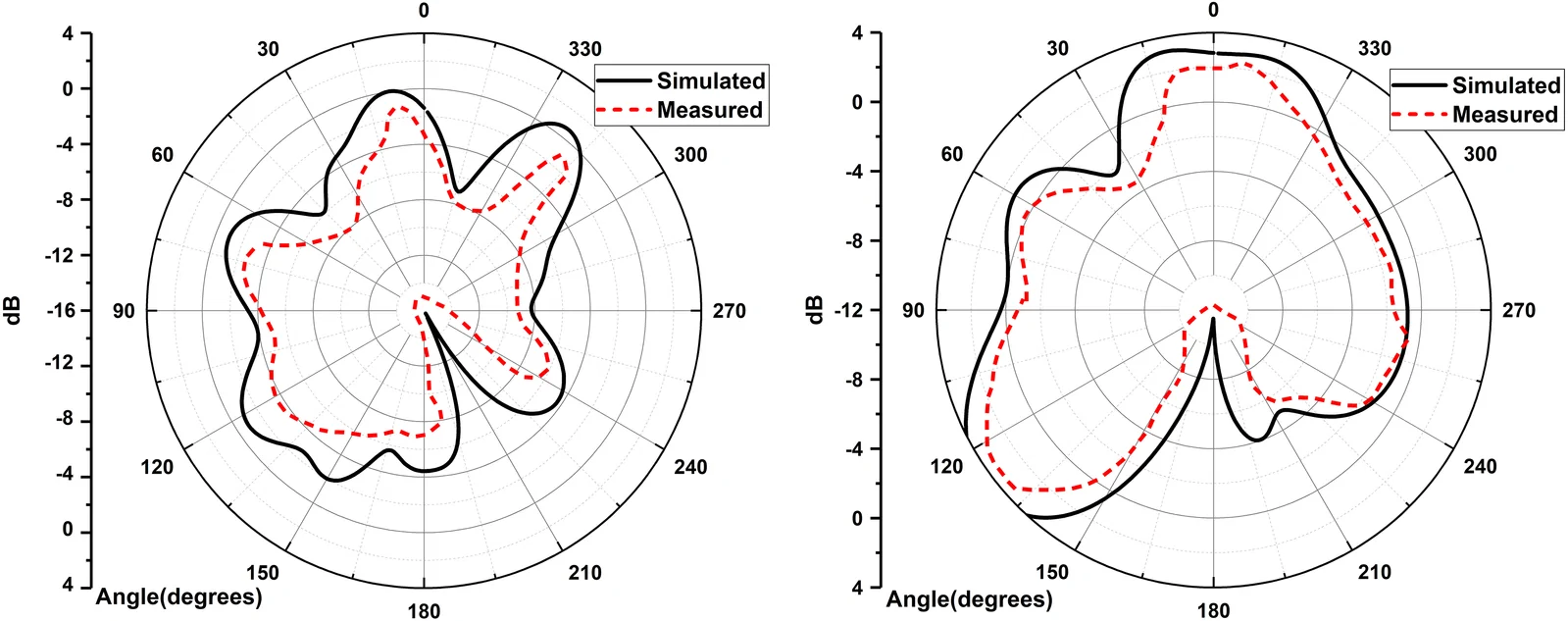
The radiation patterns (measured and simulated): Cross pol theta at 26.73 GHz and Cross pol theta at 38.29 GHz.

The proposed 4-port MIMO antenna is assessed for its spatial diversity and multiplexing performance, utilizing critical metrics like ECC, DG, TARC, MEG, CCL, and multiplexing efficiency to ensure highly reliable, high-throughput data transmission. These parameters verify that the design mitigates multipath fading and minimizes signal correlation between ports for optimal multi-antenna performance.

In a multi-channel communication system, the envelope correlation coefficient (ECC) of MIMO antennas is a crucial parameter for evaluating the level of channel isolation. The ECC should have a value of less than 0.5. The lower value of ECC signifies minimal signal interference between ports, high data capacity, and reliable multi-path performance. The S parameters using equation (1) [25] and the far-field radiation pattern using equation (2) [26] are the two methods used to get the correlation coefficient.


$$\begin{array}{c} {\text{ECC}}_{\text{i},\text{j}}=\frac{{\left|{\text{S}}_{\text{ii}}^{*}{\text{S}}_{\text{ij}}+{\text{S}}_{\text{ji}}^{*}{\text{S}}_{\text{jj}}\right|}^{\text{2}}}{\left(\text{1}-{\left|{\text{S}}_{\text{ii}}\right|}^{\text{2}}-{\left|{\text{S}}_{\text{ji}}\right|}^{\text{2}}\right)\left(\text{1}-{\left|{\text{S}}_{\text{jj}}\right|}^{\text{2}}-{\left|{\text{S}}_{\text{ij}}\right|}^{\text{2}}\right)}\; \end{array}$$ (1)

$$\begin{array}{c} {\text{ECC}}_{\text{i},\text{j}}=\frac{{\left|\iint \text{4}\pi \left[\overset{⇀}{{\text{E}}_{\text{i}}}(\theta ,\varphi ).\overset{⇀}{{\text{E}}_{\text{j}}}(\theta ,\varphi )\text{d}\Omega \right]\right|}^{\text{2}}}{\iint \text{4}\pi {\left|\overset{⇀}{{\text{E}}_{\text{i}}}(\theta ,\varphi )\right|}^{\text{2}}\text{d}\Omega \iint \text{4}\pi {\left|\overset{⇀}{{\text{E}}_{\text{j}}}(\theta ,\varphi )\right|}^{\text{2}}\text{d}\Omega } \end{array}$$ (2)

To achieve optimal diversity performance, the ECC between the MIMO antenna elements is recommended be smaller than 0.05. In the two operating frequency bands, the maximum (simulated) ECC of this antenna [Fig 27] is within 0.0003, which fulfill the required acceptable limit of ECC. By utilizing diversity technology, MIMO antennas increase the system’s signal-to-noise ratio in comparison to single antennas. The diversity gain (DG) is directly related to the ECC and can be computed using the following expression (3) [27]:

**Fig 27:**
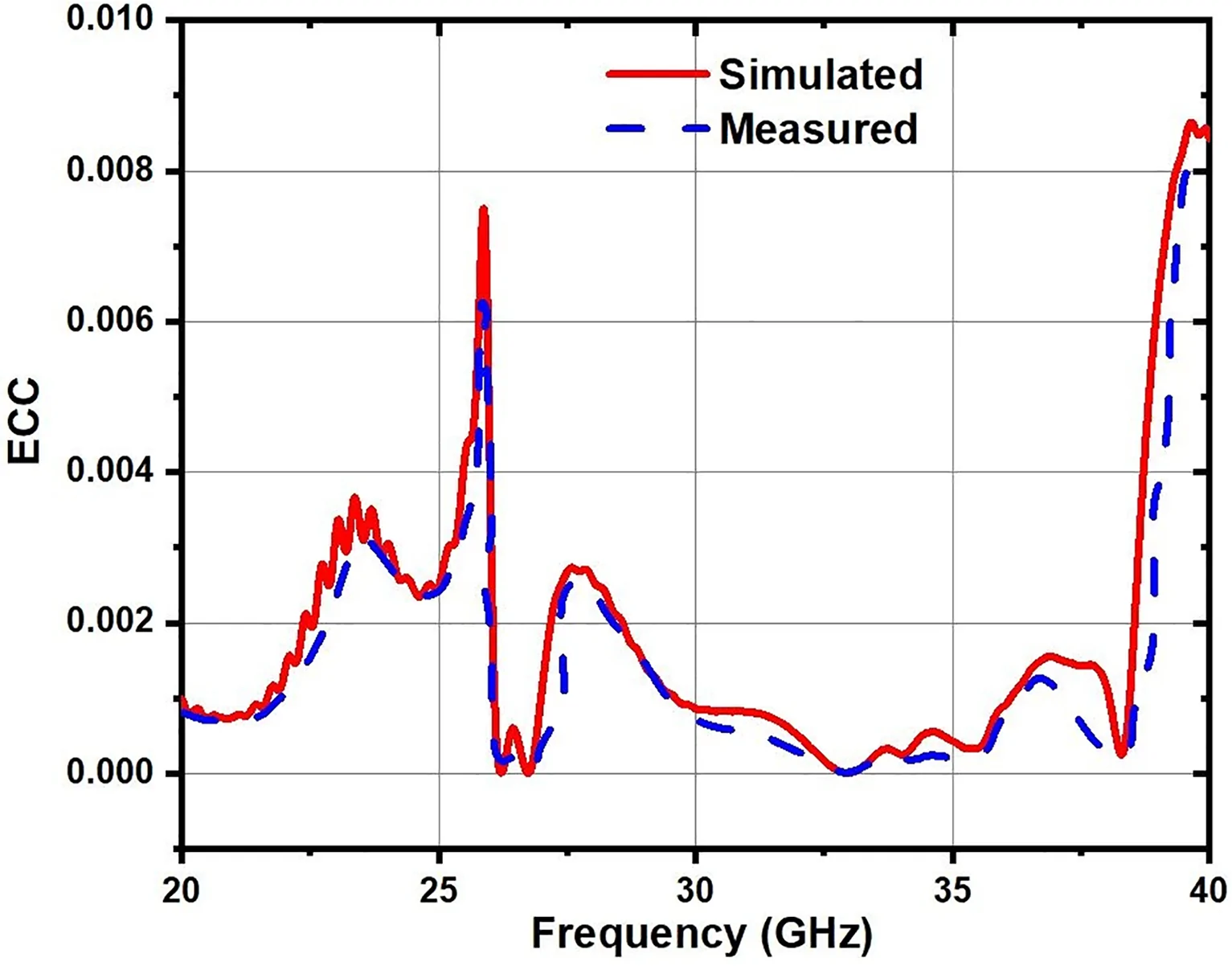
ECC vs. frequency of the proposed MIMO antenna.


$$\begin{array}{c} {\text{DG}}_{\text{i}.\text{j}}=\text{10}\sqrt{\text{1}-{\text{ECC}}_{\text{i}.\text{j}}} \end{array}$$ (3)

Fig 28 illustrates the diversity gain profile of the presented 4-port MIMO model. In comparison to the optimum DG value of 10 dB, the measured values are calculated above 9.999 dB within the targeted ranges.

**Fig 28:**
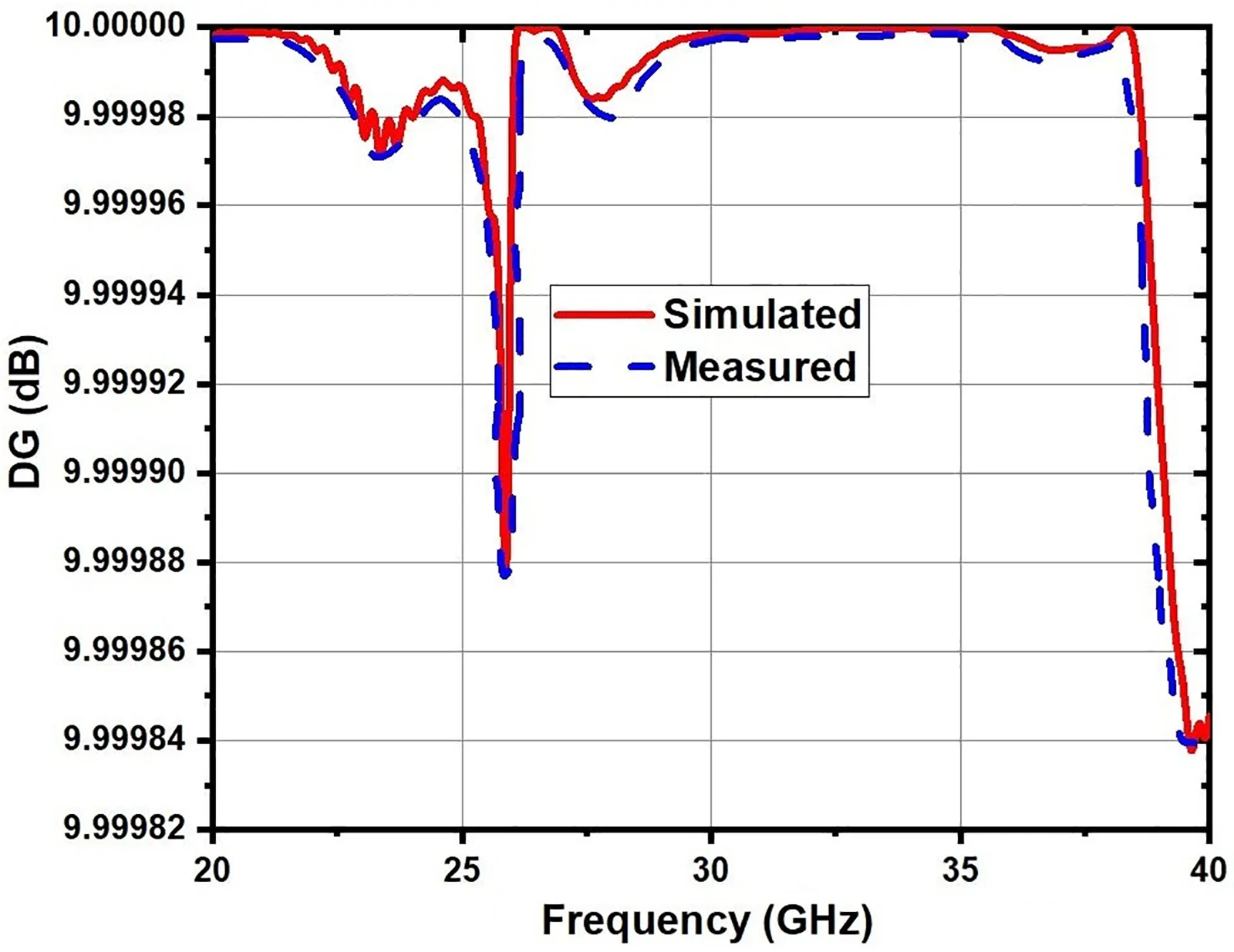
DG vs. frequency of the proposed MIMO antenna.

Channel Capacity Loss (CCL) is a critical metric in MIMO antenna design that quantifies the loss of data transmission capacity due to signal correlation and elemental coupling. In practical MIMO wireless systems, regulatory and industry standards dictate that the CCL for the functional bands should remain well below 0.5 bits/s/Hz to limit capacity degradation caused by spatial correlation and mutual coupling. Fig 29 demonstrates that the CCL for this proposed design stays within 0.1 bits/s/Hz across both functional bands (26.39 to 27.04 GHz and 38.11 to 38.47 GHz). These results indicate highly efficient data transmission and minimal information loss.

**Fig 29:**
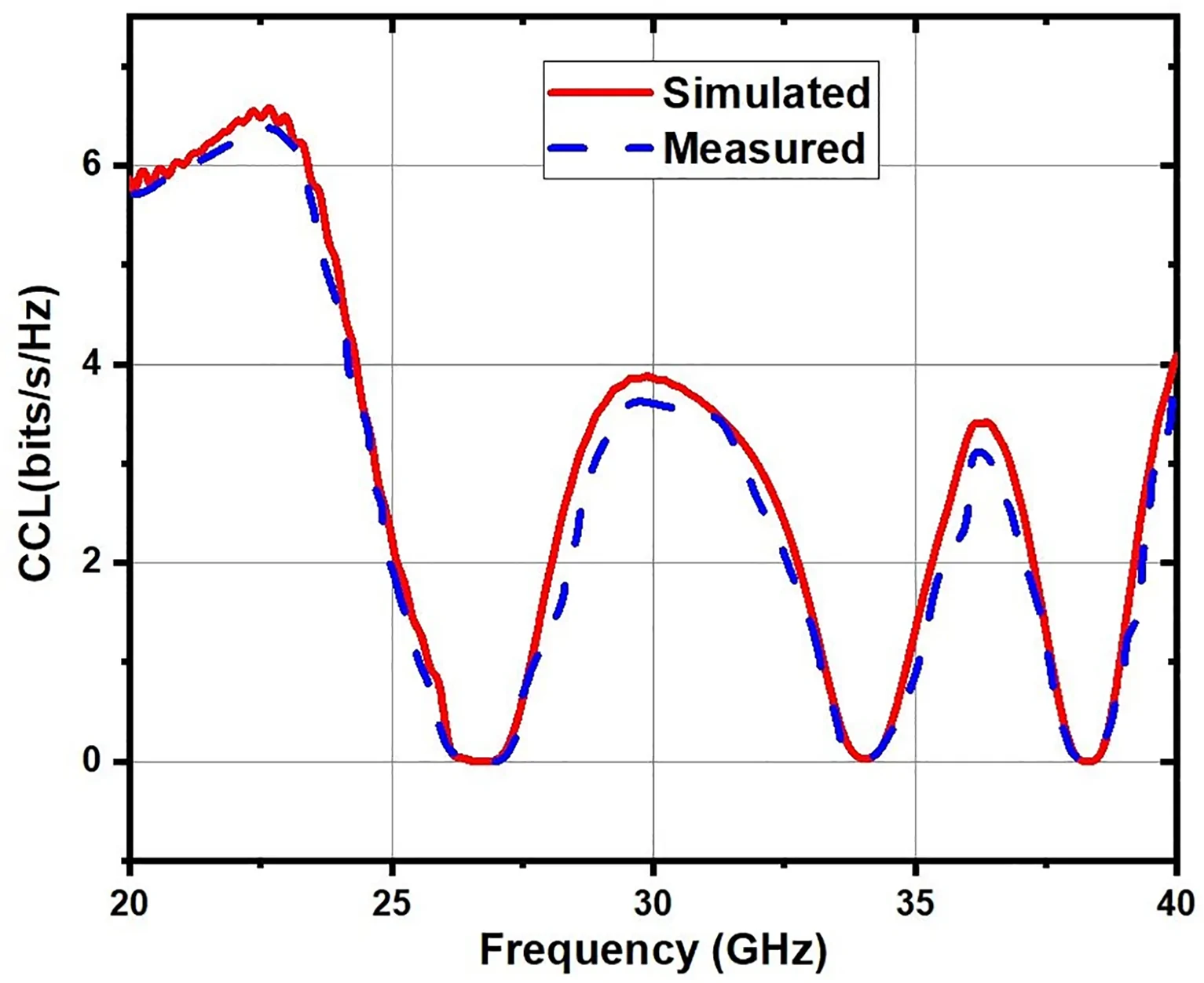
CCL vs. frequency of the proposed MIMO antenna.

The channel capacity loss is determined employing equations (4,5) [28]


$$\begin{array}{c} {\text{C}}_{\text{loss}}=-{\text{log}}_{\text{2}}\text{ det}\left(\alpha^{\text{R}}\right) \end{array}$$ (4)

$$\begin{array}{c} \alpha^{\text{R}}=\left[\begin{array}{c} \alpha_{\text{11}}\alpha_{\text{12}}\alpha_{\text{13}}\alpha_{\text{14}}\\ \alpha_{\text{21}}\alpha_{\text{22}}\alpha_{\text{23}}\alpha_{\text{24}}\\ \alpha_{\text{31}}\alpha_{\text{32}}\alpha_{\text{33}}\alpha_{\text{34}}\\ \alpha_{\text{41}}\alpha_{\text{42}}\alpha_{\text{43}}\alpha_{\text{44}} \end{array}\right] \end{array}$$ (5)

Where $\alpha_{\text{ii}}=\text{1}-\left(\sum_{\text{j}=\text{1}}^{\text{N}}{\left|{\text{S}}_{\text{ij}}\right|}^{\text{2}}\right)$ and$\alpha_{\text{ij}}=-({{\text{S}}_{\text{ij}}}^{*}{\text{S}}_{\text{ij}}+{{\text{S}}_{\text{ji}}}^{*}{\text{S}}_{\text{ij}})$

Total Active Reflection Coefficient (TARC) is a vital metric that quantifies how much incident power is reflected back due to impedance mismatch**.** A lower TARC value corresponds to less reflected power and optimal use of the delivered input power. The TARC can be computed through equation (6) [28].


$$\begin{array}{c} \text{TARC}=\frac{\sqrt{\sum_{i=\text{1}}^{N}\left|S_{i\text{1}}+\sum_{m=\text{2}}^{N}S_{im}e^{j\theta_{m-\text{1}}}\right|}}{\sqrt{N}} \end{array}$$ (6)

The expression of TARC parameter for a 4-port MIMO antenna is provided using equation (7):


$$\begin{array}{c} 𝐓𝐀𝐑𝐂=\frac{\sqrt{\begin{array}{c} {\left|{𝐒}_{11}+{𝐒}_{12}{𝐞}^{𝐣\theta_{1}}+{𝐒}_{13}{𝐞}^{𝐣\theta_{2}}+{𝐒}_{14}{𝐞}^{𝐣\theta_{3}}\right|}^{2}\\ +{\left|{𝐒}_{21}+{𝐒}_{22}{𝐞}^{𝐣\theta_{1}}+{𝐒}_{23}{𝐞}^{𝐣\theta_{2}}+{𝐒}_{24}{𝐞}^{𝐣\theta_{3}}\right|}^{2}\\ +{\left|{𝐒}_{31}+{𝐒}_{32}{𝐞}^{𝐣\theta_{1}}+{𝐒}_{33}{𝐞}^{𝐣\theta_{2}}+{𝐒}_{34}{𝐞}^{𝐣\theta_{3}}\right|}^{2}\\ +{\left|{𝐒}_{41}+{𝐒}_{42}{𝐞}^{𝐣\theta_{1}}+{𝐒}_{43}{𝐞}^{𝐣\theta_{2}}+{𝐒}_{44}{𝐞}^{𝐣\theta_{3}}\right|}^{2} \end{array}}}{\sqrt{4}} \end{array}$$ (7)

The TARC plot in Fig 30 signifies the reflection performance of the four-port MIMO radiator. A TARC value of <−10 dB is the standard threshold used to define acceptable operational bandwidth in MIMO systems, signifying that 90% of the incident power is radiated. The TARC remains lesser than −10 dB at both operating frequencies, exhibiting minimal variation with changes in input. For first operating band, the resonance dip is observed at 26.73 GHz. Both simulated and measured traces drop well below −10 dB (with the simulated curve reaching down to about −21.03 dB). For second operating band, the sharpest dip is at the resonant frequency (38.29 GHz). Both curves dip below the −10 dB threshold line, confirming that the radiator meets the operational bandwidth requirements for this higher frequency band.

**Fig 30:**
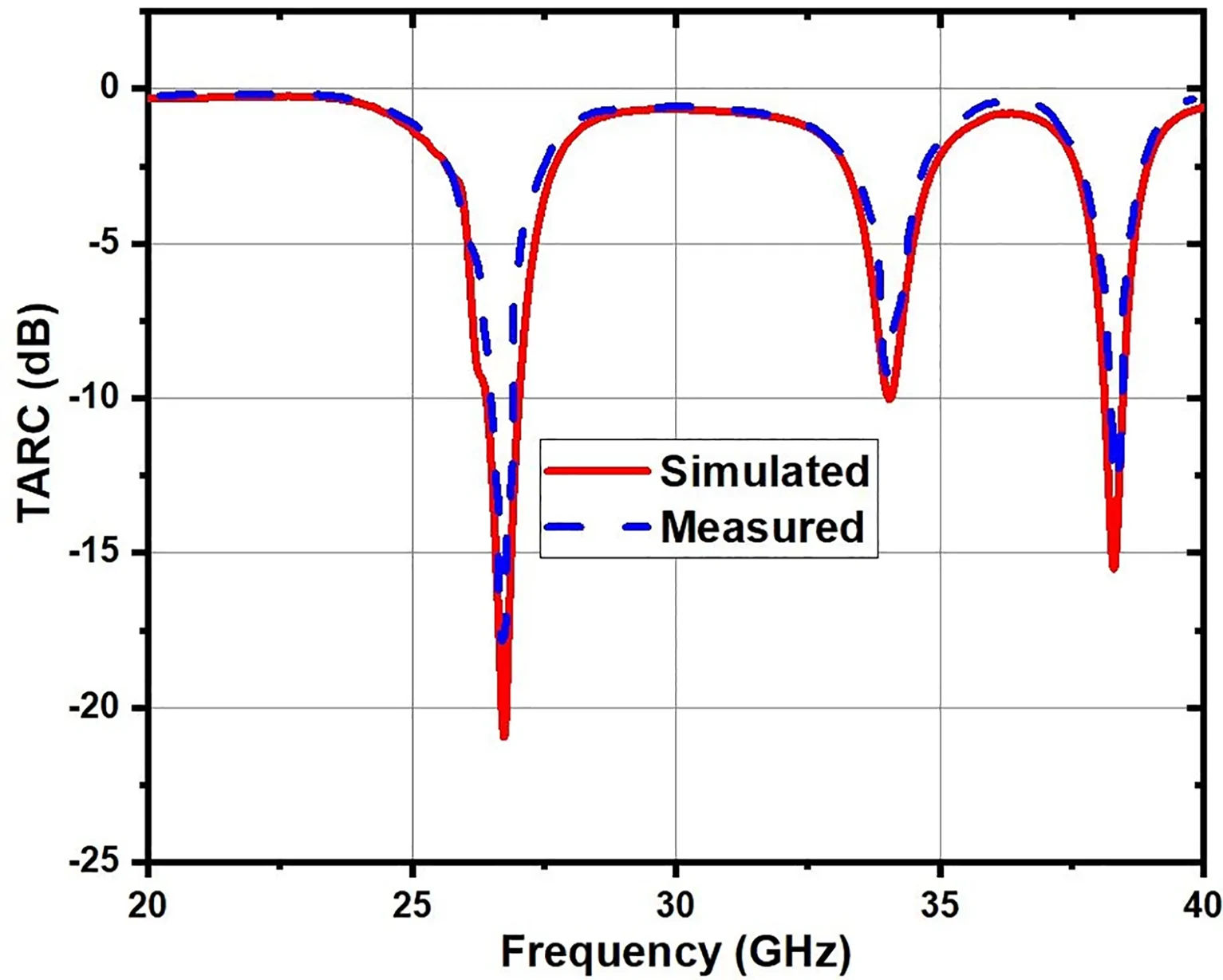
TARC vs. frequency of the proposed MIMO antenna.

The mean effective gain (MEG) indicates the MIMO radiator’s capacity to intercept EM power under rich multipath fading conditions. For robust and balanced diversity performance, the calculated MEG value should typically stay below −3 dB. It can be expressed using equation (8) [28].


$$\begin{array}{c} MEG_{i}=\frac{\text{1}}{\text{2}}\left(\text{1}-\sum_{j=\text{1}}^{\text{4}}{\left|S_{ij}\right|}^{\text{2}}\right) \end{array}$$ (8)

Where i and j indicates the port indices related to the four-port MIMO antenna system. The MEG quantity vs frequency plot for the recommended MIMO radiator is shown in Fig 31 with a solid red line representing the simulated performance, and a dashed blue line is indicating the measured prototype performance. The antenna maintains mean effective gain of less than −3 dB throughout the entire analyzed range of operation, indicating balanced power reception and efficient performance under standard conditions.

**Fig 31:**
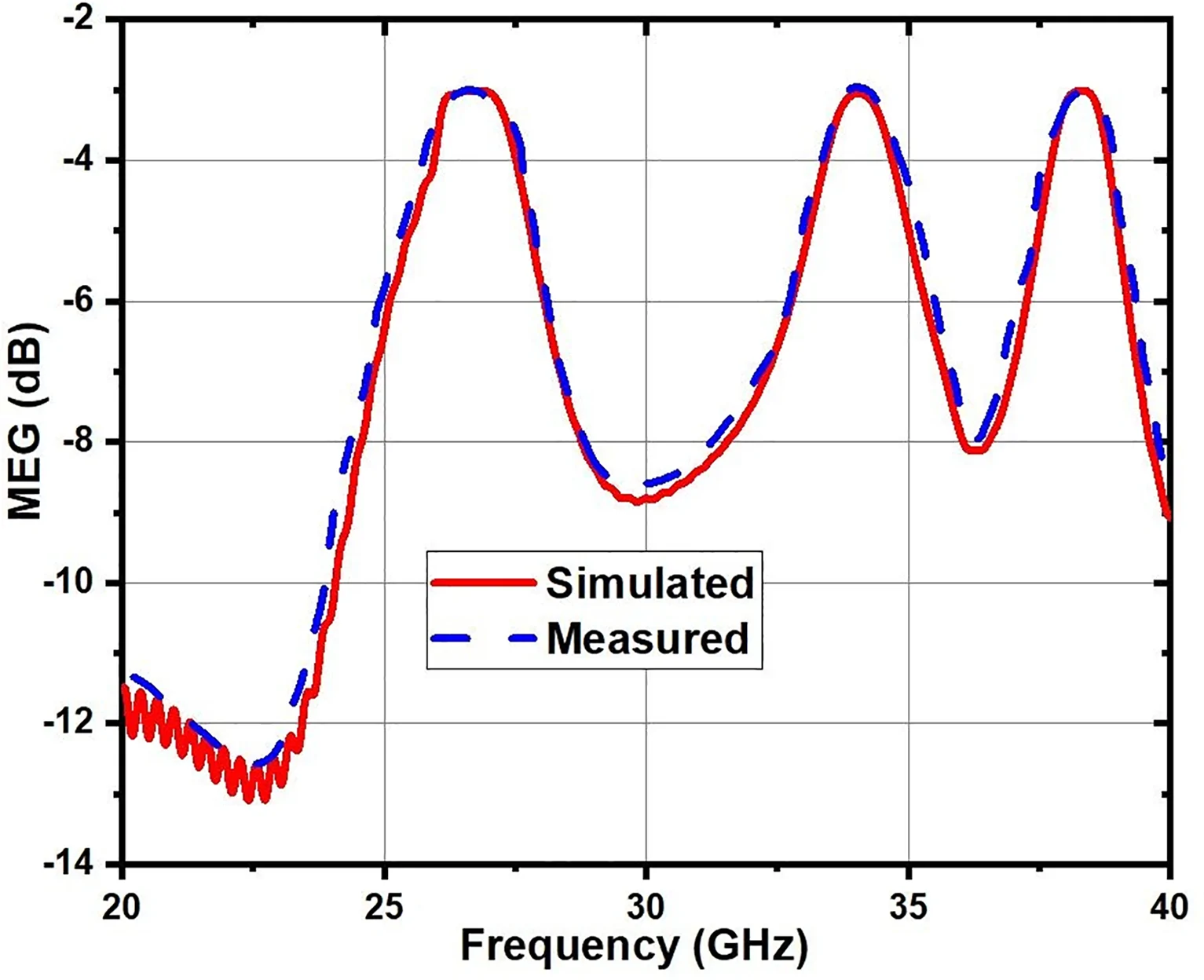
MEG vs. frequency of the proposed MIMO antenna.

The multiplexing efficiency quantifies the signal degradation caused by both power imbalances and spatial correlation between antenna elements. Fig 32 illustrates the multiplexing efficiency (%) against the frequency spectrum spanning from 24 GHz to 40 GHz. A direct comparison is presented between the full-wave EM Simulated results (solid red line) and the results obtained from measured data (dashed blue line). The multiplexing efficiency of 97.3% (simulated) and 96.4% (measured) is achieved at first resonant frequency, 26.73 GHz. Similarly, 38.29 GHz, multiplexing efficiencies of approximately 97.1% (simulated) and 96.3% (measured) are achieved. The close similarity between the findings validates the theoretical framework and design accuracy for the excellent diversity performance. The high multiplexing efficiency indicates low correlation and power loss between antenna elements, ensuring high data rates and reliable transmission with minimal channel capacity loss. The multiplexing efficiency ($\eta_{mux}$) can be calculated using the analytical expression provided in equation (9) [29].

**Fig 32:**
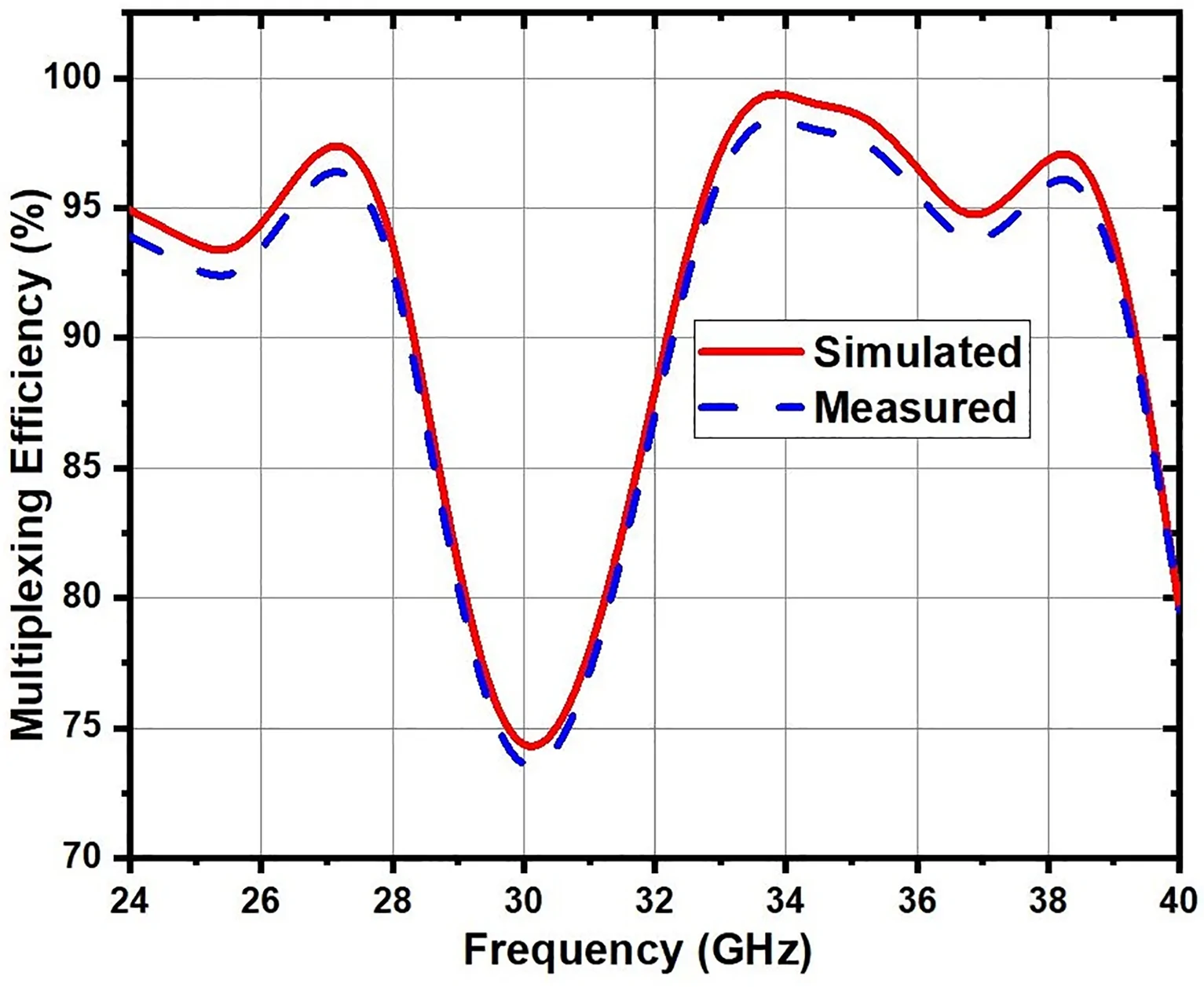
Multiplexing efficiency vs. frequency of the proposed MIMO antenna.


$$\begin{array}{c} \eta_{mux}=\sqrt[{M}]{\left(\text{1}-{\left|\rho_{C}\right|}^{\text{2}}\right)e_{tot\text{1}}\times e_{tot\text{2}}\times \ldots ..e_{totM}} \end{array}$$ (9)

Where, M = No. of antenna ports; $\rho_{c}$ = ECC between the antenna elements; $e_{tot}$ = total radiation efficiency of each individual antenna element.

Table 6 summarizes all the crucial performance parameters (both simulated and measured) of the suggested four-port MIMO radiator at its dual operating bands. The measured resonant frequencies, reflection coefficient, isolation, gain, radiation efficiency, ECC, DG, TARC, MEG, CCL, and multiplexing efficiency show acceptable agreement with the simulation-based findings. The small discrepancies are mainly attributed to fabrication tolerances, soldering effect of connector, and measurement uncertainties at millimeter-wave frequencies.

**Table 6:** Summary of simulation and measurement results for the presented four-port dual-resonant MIMO antenna.

	Resonant frequency (GHz)			
Parameter	26.73 GHz (Simulation)	26.73 GHz (Measurement)	38.29 GHz (Simulation)	38.29 GHz (Measurement)
reflection coefficients (dB)	−40.15 to −60.94 dB	−23 to −30 dB	−16.31 to −16.80 dB	−11 to −15 dB
Isolation (dB)	> 19.32	> 19	> 22.12	> 19
Gain (dBi)	4.80	4.63	7.61	7.22
Radiation efficiency (%)	97. 61%	≈ 94.12%	95.45%	≈ 95.21%
ECC	0.0001	0.0003	0.00028	0.0004
DG (dB)	~10 dB	~9.9999 dB	~10 dB	~9.9998 dB
TARC (dB)	−21.03	−17.94	−15.42	−12.03
MEG (dB)	<−3	<−3	<−3 –	<−3
CCL (bits/s/Hz)	<0.1	<0.1	<0.1	<0.1
Multiplexing Efficiency (%) ($\eta_{mux}$)	97.3	96.4	97.1	96.3

To understand the physical insight behind the dual-band operation and isolation enhancement, the surface current distributions at 26.73 GHz and 38.29 GHz for different antenna ports are analyzed in Figs 33–36. At 26.73 GHz and 38.29 GHz, the antenna elements exhibit maximum surface current amplitudes of 260 A/m and 1092 A/m, respectively. These values are derived from the surface current distribution, which includes a corresponding color scale. In general, the concentration of the current distributions is observed along the margins of the patches and in the ground plane slots. On the surface of the radiating patch, current distributions are found to be larger in the circular and trapezoidal DGS slots. This indicates that the slots have improved the bandwidth of the antenna by facilitating the transmission of electromagnetic waves. Specifically, at 26.73 GHz Fig 33, the surface current flows uniformly along the entire length of the patch, signifying that the fundamental mode governs the lower band. Conversely, at 38.29 GHz, intense current concentration shifts towards the slot edges and curvilinear discontinuities, generating a secondary shorter effective path that yields the upper resonant band. Furthermore, the decoupling mechanism introduced by the trapezoidal-shaped based defected ground structure (DGS) is apparent in minimizing mutual coupling among orthogonally placed antenna elements. When an individual port is active, the DGS behaves as a high-impedance band-stop wall. It traps and perturbs the ground plane currents, preventing them from coupling into adjacent elements. This causes the unexcited patches to exhibit minimum current intensity, which directly supports the highly suppressed mutual coupling observed in the S-parameter characteristics.

**Fig 33:**
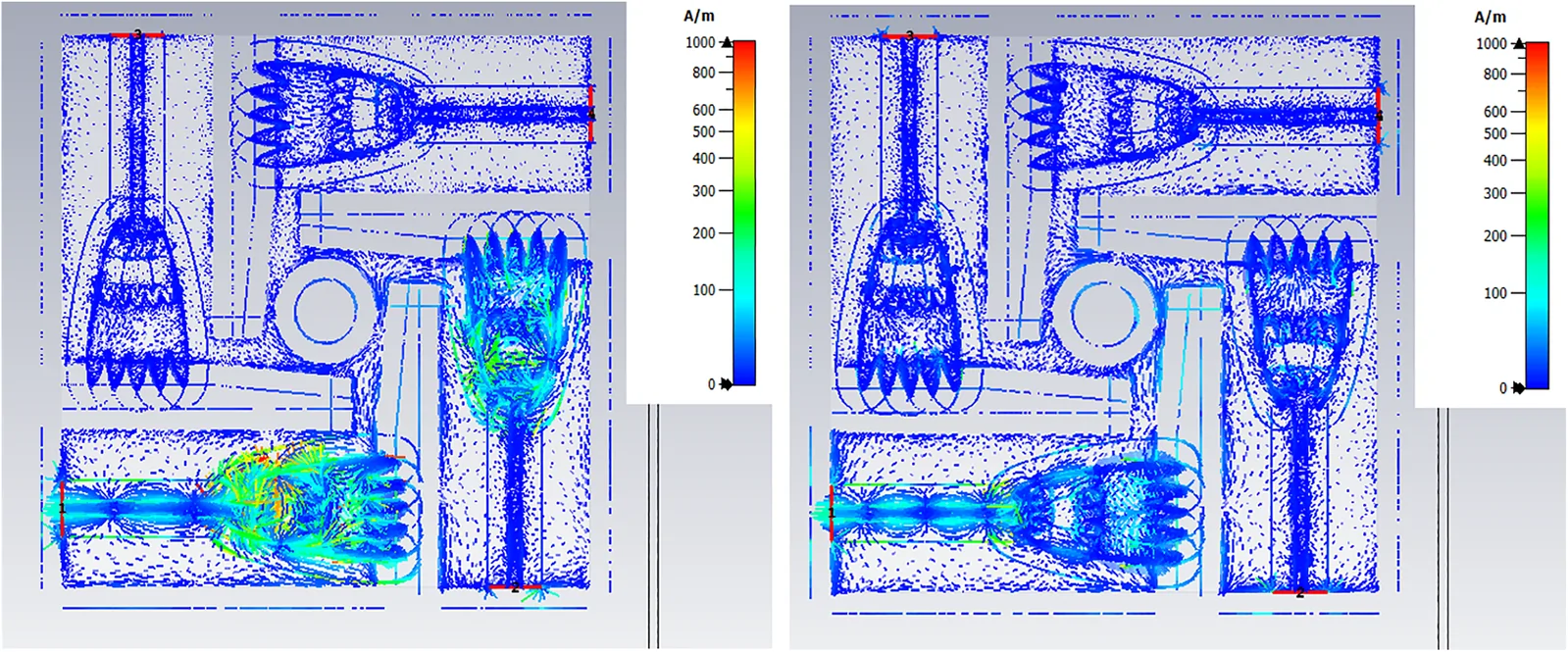
The surface current distributions for port 1 at 26.73 GHz and 38.29 GHz.

**Fig 34:**
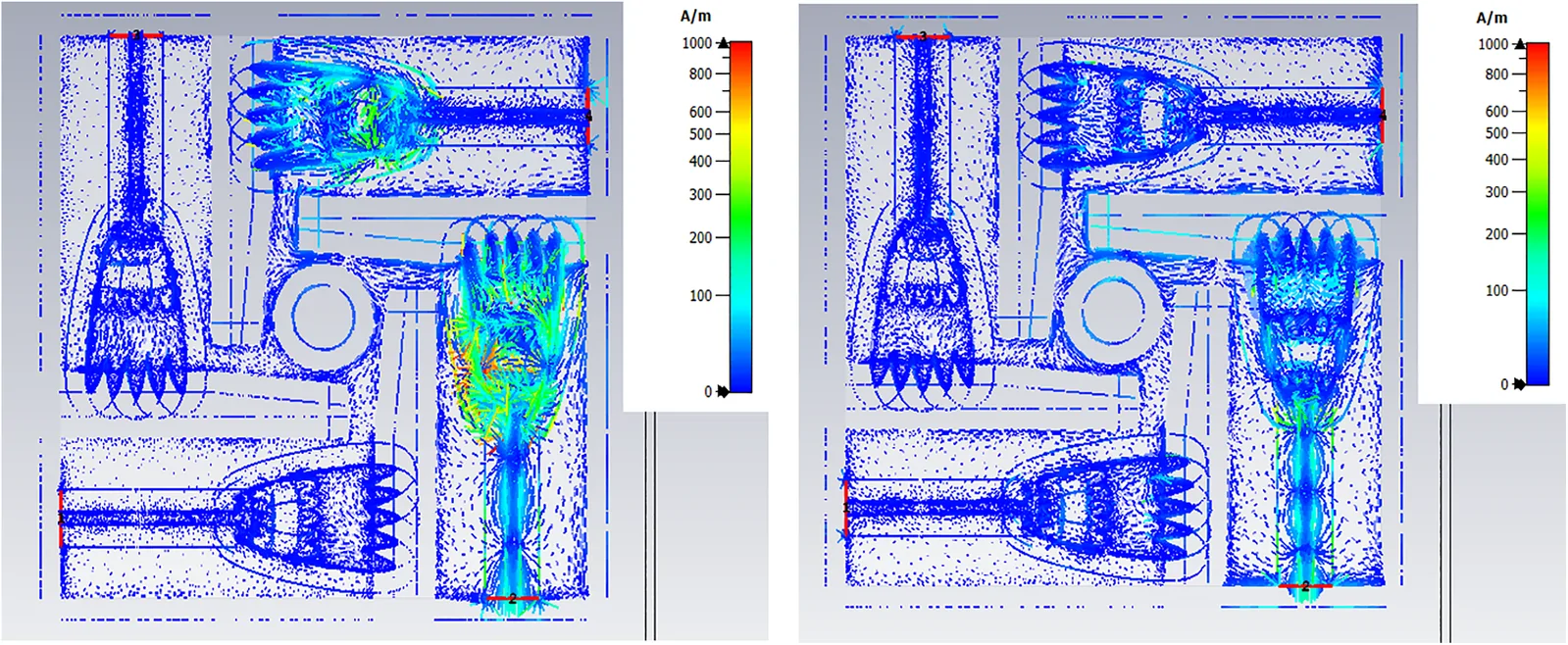
The surface current distributions for port 2 at 26.73 GHz and 38.29 GHz.

**Fig 35:**
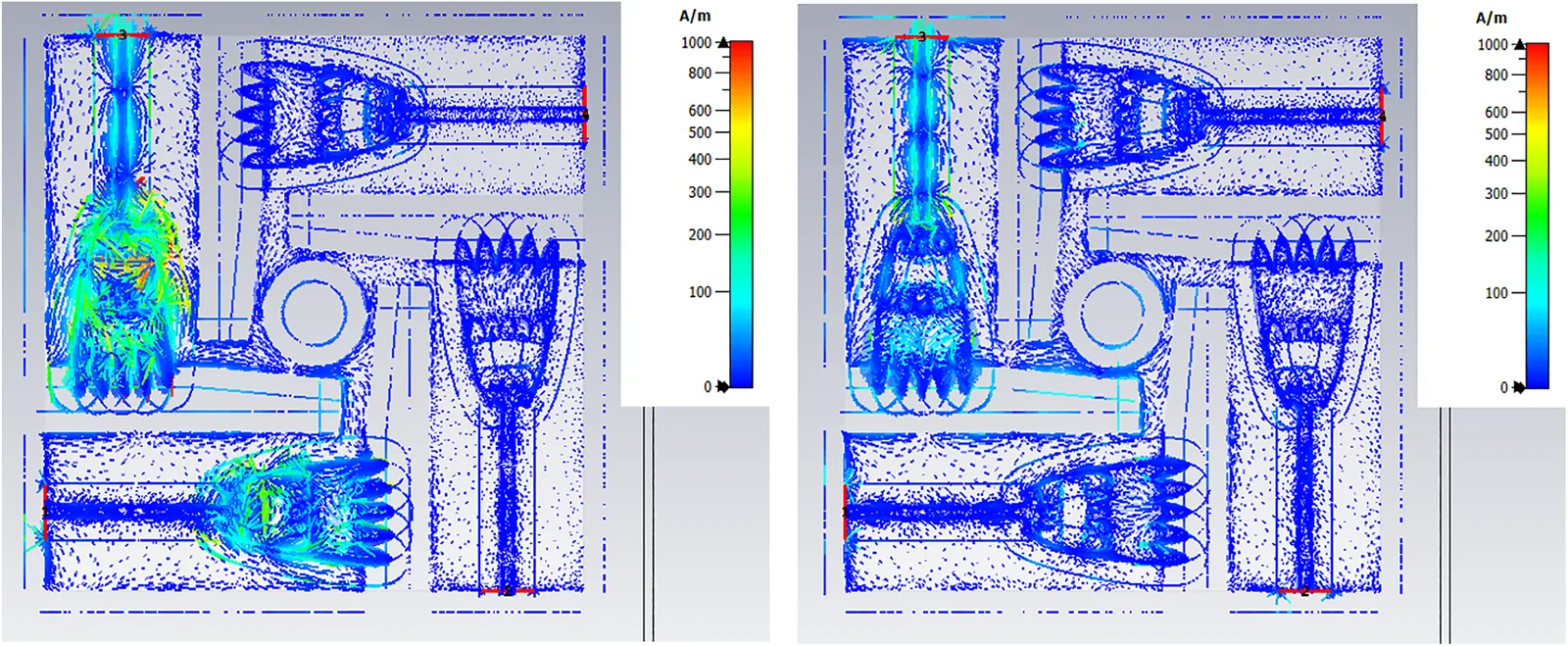
The surface current distributions for port 3 at 26.73 GHz and 38.29 GHz.

**Fig 36:**
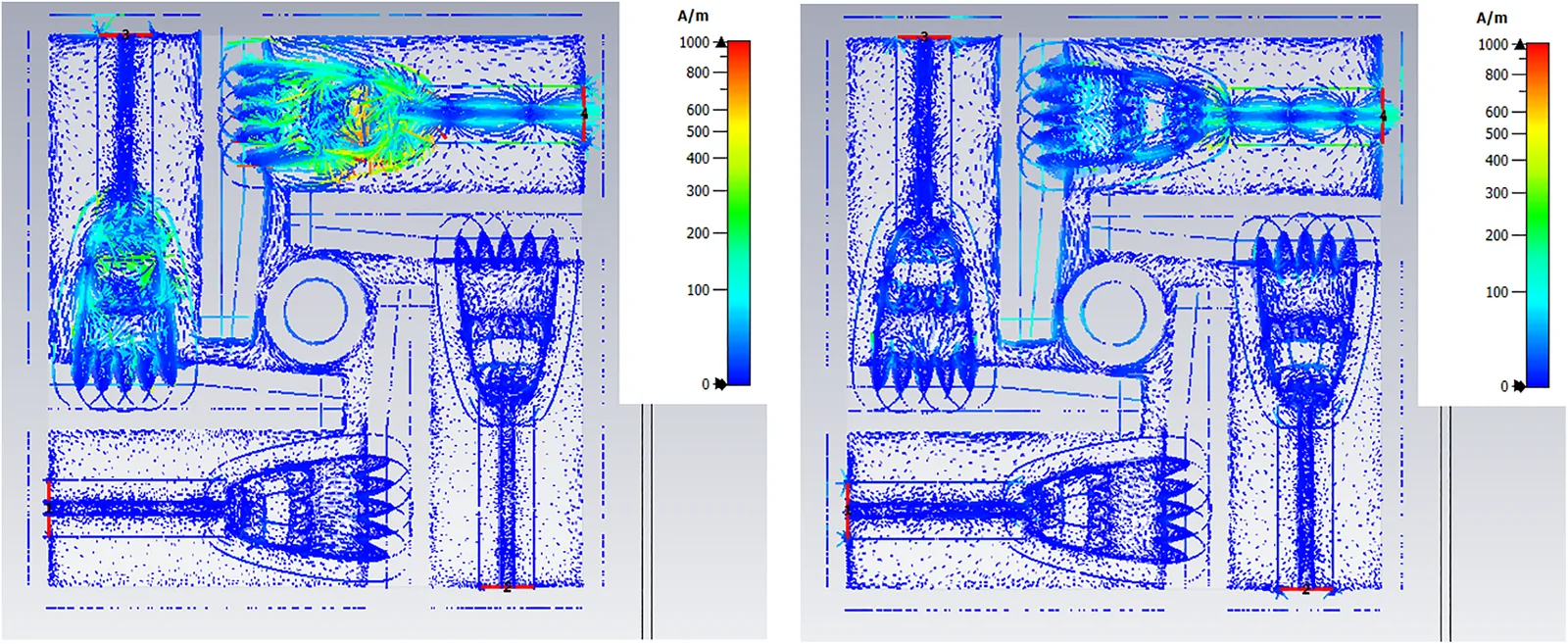
The surface current distributions for port 4 at 26.73 GHz and 38.29 GHz.

## 6. Performance comparison analysis

Table 7 shows a summary of the performance comparison with other reported MIMO designs [12,13,17–23]. The MIMO antenna analysis has been justified by including details of designed methodology and comparing various important characteristics such as their physical size, electrical size with respect to lowest operating frequency, number of ports used in antenna configurations, resonant frequency, impedance bandwidth, gain, radiation efficiency, gain-to-area ratio, reflection coefficient, isolation and radiation efficiency, and various MIMO diversity parameters. Unlike previously reported configurations, which evaluate only a subset of MIMO metrics, this study provides an exhaustive investigation of all diversity parameters: CCL, MEG, TARC, and multiplexing efficiency along with ECC and DG discussed in results and discussion section. The proposed antenna system yields highly favorable values across all evaluated metrics, ensuring robust real-world performance. Furthermore, the suggested antenna possesses highly competitive impedance and radiation performance with a compact physical footprint and electrical size in comparison to the state-of-the-art. These collective features make the suggested hybrid DGS-based four-port MIMO design a highly suitable choice for intended dual band 5G applications in mmWave range.

**Table 7:** Comparative outline of the recommended MIMO antenna with other antennas documented in the literature.

Ref.	Antenna geometry/ Physical Size in (mm^2^)/ Electrical size in terms of λ	No. of ports	Gain (dBi)/ Rad. Efficiency (%)	$f_{r}$ (GHz)/ BW (GHz)	S_ii_ (dB)	Gain-to-Area Ratio	Isolation (dB)	TARC (dB)	MEG (dB)	CCL (bits/s/Hz)	$\eta_{mux}$ (%)
[12]	MIMO with FSS/ (36.2 × 36. 2)/ (4.49λ × 4.49λ)	4	8.2-10/82	38/2	−24	0.00625-0.00763	>25	NP	NP	<0.4	NP
[13]	MIMO with semicircular slots based square DGS/ 25 × 25 (2.21λ × 2.21λ)	4	8.72/NP	26.26/2.74	−23.5	0.01395	>23.5	−29	NP	NP	NP
[18]	Modified tapered patch with partial ground plane/ 26 × 14 (2.31λ × 1.24λ)	2	NR	NR	−22	NR	>20	NP	NP	<0.4	NP
			NR	NR	−15	NR	>20	NP	NP	<0.4	NP
[19]	Phased array MIMO patch with EBG cells/ 26 × 19.25 (2.342λ × 1.734λ)	4	7.58 dB/ > 86	28/ 1.44	−40.35	0.01514	>20	NP	NP	NP	NP
			5.72 dB/ > 86	38/0.35	−38.80	0.01142	>20	NP	NP	NP	NP
[20]	Slot-based triangular extended monopole/ 26 × 11 (2.255λ × 0.954λ)	2	5/99.5	27/3	−22.5	0.01748	>30	NP	NP	NP	NP
			5.7/98.6	39/5	−27.5	0.01993	>25	NP	NP	NP	NP
[17]	MIMO array with T-junction divider and different types of DGS 35 × 30 (2.976λ × 2.551λ)	4	8.3/82	28/4.1	-----	0.00790	>15	NP	NP	<0.4	NP
[21]	Metamaterials integrated modified patch and ground plane 18 × 9.2 (1.44λ × 0.736λ)	2	7.8/NP	28/4.8	NP	0.04710	30	NP	NP	0.05	NP
			6.0/NP	38/4.2	NP	0.03623	28	NP	NP	0.05	NP
[22]	Composite patch with slots-based ground plane 18 × 8.5 (1.665λ × 0.786λ)	4	4.15/80.13	28/0.72	–18.5	0.02712	>20	−25	− 6	< 0.15	NP
			7.73/85.44	38/0.5	–21.1	0.05052	>15	−34	− 6	< 0.15	NP
[23]	Crescent shaped monopole 20 × 20 (1.801λ × 1.801λ)	4	9.5/ > 80	28/2	<–20	0.02375	>21.5	NP	NP	<0.1	NP
			11.7/ > 80	38/6.5	<–33	0.02925	>22.5	NP	NP	<0.1	NP
**Proposed**	Flower-shaped elliptical patch with hybrid DGS 21 × 21 (1.849λ × 1.849λ)	4	4.80/97.61	26.73/0.65	−60.94	0.01088	>19.32	−21.03	<−3	<0.1	97.3
			7.61/95.45	38.29/0.36	−16.80	0.01725	>22.12	−15.42	<−3	<0.1	97.1

NR = Not provided.

## 7. Conclusion

The use of quadri-port MIMO antennas in 5G networks is essential to meet the growing demand for faster data rates, lower latency, and the ability to connect a larger number of devices. The recommended MIMO antenna is designed using a modular approach, starting from a single elliptical patch element, two-element MIMO, and extending to a four-element MIMO configuration to provide improved diversity performance. A novel hybrid defected ground structure (DGS) is introduced to enhance isolation and minimize coupling, while maintaining compact dimensions of 21 × 21 mm^2^. The proposed model shows dual-band resonance with a gain of 7.22 dBi at upper resonant frequency, and maximum radiation efficiency of 97.61% at 26.73 GHz. Furthermore, the presented four-port radiator demonstrates exceptional diversity performance, proving its efficacy in mitigating multipath fading and ensuring reliable coverage. These experimental findings demonstrate the proposed design to be a promising option for next-generation 5G mmWave applications.

## References

[1] Tran-Tuan A, Hoang-Thi-Phuong T, Vu-Van Y, Tran-Huy H. A simple technique to improve performance of four-port wideband MIMO antenna using shorting pins. PLoS One. 2025;20(5):e0321602. doi: 10.1371/journal.pone.0321602 40408326 PMC12101636

[2] Ishteyaq I, Muzaffar K. Multiple input multiple output (MIMO) and fifth generation (5G): An indispensable technology for sub-6 GHz and millimeter wave future generation mobile terminal applications. Int J Microw Wireless Technol. 2021;14(7):932–48. doi: 10.1017/s1759078721001100

[3] Ziemer RE. An Overview of Millimeter Wave Communications. In: 14th European Microwave Conference, IEEE, 1984. p. 3–8. 10.1109/euma.1984.333326

[4] Alkhateeb A, El Ayach O, Leus G, Heath RW. Channel estimation and hybrid precoding for millimeter wave cellular systems. IEEE J Sel Top Signal Process. 2014;8(5):831–46. doi: 10.1109/jstsp.2014.2334278

[5] Rangan S, Rappaport TS, Erkip E. Millimeter-wave cellular wireless networks: potentials and challenges. Proc IEEE. 2014;102(3):366–85. doi: 10.1109/jproc.2014.2299397

[6] Soman M, Sharma M, Narwal S, Ali T. Eight-Port millimeter wave MIMO antenna loaded with novel frequency selective surface for higher data rate transmission. PLoS One. 2026;21(2):e0341823. doi: 10.1371/journal.pone.0341823 41628194 PMC12863549

[7] Raj T, Mishra R, Kumar P, Kapoor A. Advances in MIMO antenna design for 5G: A comprehensive review. Sensors (Basel). 2023;23(14):6329. doi: 10.3390/s23146329 37514623 PMC10384345

[8] Liu X, Zhang J, Xi H, Yang X, Sun L, Gan L. A compact four-band high-isolation quad-port MIMO antenna for 5G and WLAN applications. AEU - Int J Electronics Commun. 2022;153:154294. doi: 10.1016/j.aeue.2022.154294

[9] El Hadri D, Zakriti A, Zugari A, El Ouahabi M, El Aoufi J. High isolation and ideal correlation using spatial diversity in a compact MIMO antenna for fifth-generation applications. Int J Antennas and Propagation. 2020;2020:1–10. doi: 10.1155/2020/2740920

[10] Mchbal A, Touhami NA, Elftouh H, Moubadir M, Dkiouak A. Spatial and polarization diversity performance analysis of a compact MIMO antenna. Procedia Manufacturing. 2019;32:647–52. doi: 10.1016/j.promfg.2019.02.266

[11] Umamaheshwar S, Mahender K, Gopal M. Novel hybrid MIMO detector for spatial multiplexed MIMO system. IOP Conf Ser: Mater Sci Eng. 2020;981(3):032039. doi: 10.1088/1757-899x/981/3/032039

[12] Ibrahim AA, Ali WAE, Alathbah M, Sabek AR. Four-Port 38 GHz MIMO antenna with high gain and isolation for 5G wireless networks. Sensors (Basel). 2023;23(7):3557. doi: 10.3390/s23073557 37050618 PMC10099217

[13] Güler C, Bayer Keskin SE. A novel high isolation 4-port compact MIMO antenna with DGS for 5G applications. Micromachines (Basel). 2023;14(7):1309. doi: 10.3390/mi14071309 37512619 PMC10386084

[14] Khandelwal MK, Kanaujia BK, Kumar S. Defected ground structure: fundamentals, analysis, and applications in modern wireless trends. Int J Antennas and Propagation. 2017;2017:1–22. doi: 10.1155/2017/2018527

[15] Guha D, Biswas S, Antar YMM. Defected Ground Structure for Microstrip Antennas. Microstrip and Printed Antennas. Wiley. 2010. 387–434. doi: 10.1002/9780470973370.ch12

[16] Xing H, Wang X, Gao Z, An X, Zheng H, Wang M, et al. Efficient Isolation of an MIMO Antenna Using Defected Ground Structure. Electronics. 2020;9(8):1265. doi: 10.3390/electronics9081265

[17] Khalid M, Iffat Naqvi S, Hussain N, Rahman M, Fawad, Mirjavadi SS, et al. 4-Port MIMO antenna with defected ground structure for 5G millimeter wave applications. Electronics. 2020;9(1):71. doi: 10.3390/electronics9010071

[18] Hasan MN, Bashir S, Chu S. Dual band omnidirectional millimeter wave antenna for 5G communications. J Electromagnetic Waves and Appl. 2019;33(12):1581–90. doi: 10.1080/09205071.2019.1617790

[19] Thi Thanh Tu D, Gia Thang N, Tuan Ngoc N, Thi Bich Phuong N, Van Yem V. 28/38 GHz dual-band MIMO antenna with low mutual coupling using novel round patch EBG cell for 5G applications. In: 2017 International Conference on Advanced Technologies for Communications (ATC). IEEE, 2017. p. 64–9.

[20] Ali W, Das S, Medkour Hicham H. Planar dual-band 27/39 GHz millimeter-wave MIMO antenna for 5G applications. Microsystem Technologies. 2021:27;283–92.

[21] Khan D, Ahmad A, Choi D-Y. Dual-band 5G MIMO antenna with enhanced coupling reduction using metamaterials. Sci Rep. 2024;14(1):96. doi: 10.1038/s41598-023-50446-0 38168470 PMC10761735

[22] Tiwari RN, Sharma D, Singh P. A flexible dual-band 4× 4 MIMO antenna for 5G mm-wave 28/38 GHz wearable applications. Sci Rep. 2024;14(1):14324. 10.1038/s41598-024-65023-2 PMC11192723 38906936

[23] Sghaier N, Belkadi A, Hassine IB, Latrach L, Gharsallah A. Millimeter-wave dual-band MIMO antennas for 5G wireless applications. J Infrared Milli Terahz Waves. 2023;44(3–4):297–312. doi: 10.1007/s10762-023-00914-5

[24] Anouar E-S, Bendaoued M, Rihab R. Design and analysis of a dual-band 27/38 GHz elliptical-shaped patch antenna for 5G applications. J Nano-and Electronic Physics. 2022;14(5).

[25] Arrouch TE, Das S, Langhnoja NV, Islam T, Algarni AD, El Idrissi NEA, et al. Design of a circularly polarized printed antenna and its MIMO configuration for modern Ultra‐Wideband (UWB) wireless communication systems: Simulation and experimental validation. Int J Numerical Modelling. 2026;39(3). doi: 10.1002/jnm.70173

[26] Gautam PK, Srivastava G, Jhariya DK, Mohan A, Kumar S, Pathan S. A dual-band four-port MIMO antenna with a partial ground plane for n257/n260/n261 band applications. Sci Rep. 2026;16(1):13122. doi: 10.1038/s41598-026-43355-5 41813876 PMC13100126

[27] Thanki P, Upadhyaya T, Patel U, Sorathiya V, Khishe M. Planar compact four port MIMO antenna for Ultra Wideband applications. PLoS One. 2024;19(12):e0314193. doi: 10.1371/journal.pone.0314193 39621596 PMC11611179

[28] Kiouach. A high isolation wideband palm tree-shaped printed 4 × 4 MIMO antenna for 5G mm-waves applications. AEU - Int J Electronics Commun. 2024;179;155322.

[29] Chen X. Throughput multiplexing efficiency for MIMO antenna characterization. Antennas Wirel Propag Lett. 2013;12:1208–11. doi: 10.1109/lawp.2013.2282952

