# Supplementary material for: A flower-shaped dual-band four port MIMO antenna with novel DGS for 5G mmWave applications
Source: PLoS One. 2026 Sep 28;21(9):e0358640. doi: 10.1371/journal.pone.0358640 (PMC13619068; doi:10.1371/journal.pone.0358640)
Supplement: Supplementary file 1 [file pone.0358640.s002.pdf]

Response to Respected Reviewer's Comments on Manuscript ID: PONE-D-26-17759

To
The Respected Editor in Chief
PLOS One
Thank you very much for giving us the opportunity to revise our manuscript entitled **"A Flower-shaped Dual-band Four Port MIMO Antenna with Novel DGS for 5G mm-wave (27/38 GHz) Applications" (PONE-D-26-17759)**, **"A Flower-shaped Dual-band Four Port MIMO Antenna with Novel DGS for 5G mmWave Applications"**

We would like to express our sincere gratitude to you, the Associate Editor, and the reviewers for the careful evaluation of our manuscript. We are truly grateful to Respected Editor and Reviewers for providing the constructive comments and suggestions, which helped us improve the quality, clarity, and technical depth of the work.

All the suggestions provided by the Respected Reviewers have been thoroughly addressed and incorporated into the revised manuscript. The responses to the reviewer's suggestions are also appended in this letter as follows.
The modifications made in response to the comments of Respected Reviewer1 are highlighted in **Red** and for Respected Reviewer, the suggested changes are marked in **Blue.**

We sincerely hope that the revised version satisfactorily addresses all the concerns raised during the review process.

Thanking You,
Authors of the manuscript.

---xx--

Comments from Respected Reviewers

Reviewer 1

Comments to the Author

The manuscript proposes a compact flower-shaped dual-band four-port MIMO antenna with a hybrid defected ground structure for 27/38 GHz 5G mm-wave applications. The study includes a single-element design, two-port arrangement analysis, four-port MIMO configuration, DGS parameter optimization, fabrication, and measurement. The overall topic is relevant, and the manuscript has publication potential.

However, several specific issues should be corrected before the manuscript can be accepted.

Response to Respected Reviewer 1

We would like to express our sincere gratitude to **Respected Reviewer 1** for the time and effort devoted to evaluating our manuscript, as well as for the constructive and encouraging comments

provided. We are pleased that the reviewer found the proposed topic relevant, and indicated its potential for publication. The remarks have helped us improve several important aspects of the paper. We have carefully revised the manuscript and provided detailed responses to each comment. A detailed point-by-point response is provided below, and all modifications related to **Respected Reviewer 1's** comments are marked in red in the revised manuscript.

Comment 1: Reflection-coefficient values at 27 GHz and 38 GHz are inconsistent. In the Introduction, in the paragraph beginning with "The stated objective of this research is to develop a four-element MIMO…", the manuscript states:
"The reflection coefficient drops to −16.80 dB at 27 GHz and −60.94 dB at 38 GHz."
However, this is not consistent with the parametric results in Tables IV and V.
For the final parameter set in Table IV, when hQ = 2 mm, the reported values are:
• Frequency: 26.73 GHz and 38.29 GHz
• Sii: −60.94 dB at the lower band
• Sii: −16.80 dB at the upper band
Similarly, in Table V, when WQ = 5.5 mm, the reported values are:
• Frequency: 26.74 GHz and 38.29 GHz
• Sii: −60.94 dB at the lower band
• Sii: −16.80 dB at the upper band
Therefore, the values appear to be reversed in the Introduction. Please check and correct the reported reflection-coefficient values throughout the manuscript. The final values should be consistently reported in the Introduction, Section 4, Tables IV–V, the measurement discussion, and the Conclusion.

Response: **Respected Reviewer, Thank you so much for this helpful comment.** We are extremely sorry for the unintentional typographical mistakes. We are highly thankful to the Respected Reviewer for pointing out these typing mistakes. The manuscript has been carefully revised to ensure complete consistency in writing the optimal values of reflection coefficient and operating frequencies throughout the revised manuscript (Introduction, Section 4, Tables IV–V, measurement discussion, and Conclusion) for your kind consideration. The exact optimal values are

Frequency: 26.73 GHz and 38.29 GHz with Sii: −60.94 dB at the lower band and Sii: −16.80 dB at the upper band.

Comment 2: The final optimized value of RDGS is unclear. Section 4.3 discusses the effect of the circular DGS radius RDGS. The text states:
"This indicates that the DGS radius (RDGS), RDGS = 2.6 mm, is utilized in the proposed design."
However, the numerical values discussed immediately before this sentence correspond to RDGS = 2 mm in Table III, not RDGS = 2.6 mm.
Specifically, Section 4.3 states:
• Sii = −54.79 dB at 27 GHz
• Sii = −16.59 dB at 38 GHz

• bandwidth = 0.651 GHz and 0.362 GHz
• gain = 4.73 dBi and 7.08 dBi
In Table III, these values belong to the RDGS = 2 mm column. The RDGS = 2.6 mm column gives different values:
• Sii = −38.65 dB and −16.40 dB
• bandwidth = 0.640 GHz and 0.357 GHz
• gain = 4.71 dBi and 6.72 dBi
In addition, Table II lists the final design parameter as RDGS = 2 mm. Please clarify which RDGS value was finally selected and which value was used in the fabricated prototype. The text in Section 4.3 should be corrected accordingly.
There is also a small frequency wording issue in Section 4.3: the text states "At 28 GHz, a gain of 4.73 dBi…", but the target lower band and the table values are around 26.76–27 GHz. Please check whether "28 GHz" should be revised to "27 GHz" or to the exact simulated frequency.

Response: Respected Reviewer, Thank you so much for this helpful comment. We are extremely sorry for the unintentional typographical mistakes. We are highly thankful to the **Respected Reviewer** for pointing out these mistakes. The final optimized value of the circular defected ground structure radius used in both simulation and fabrication is R_{DGS} = 2 mm, as correctly indicated in Table II. The discussion in Section 4.3 referring to R_{DGS} = 2.6 mm was a typographical error and is corrected in the revised manuscript, replacing the value by R_{DGS} = 2 mm. The variation of RDGS was studied to evaluate its effect on impedance matching, gain, and bandwidth, and the final selected value was chosen based on the best overall performance trade-off. Finally, the frequency notation in Section 4.3 has been corrected to match the exact simulated resonance frequency instead of the approximate value previously stated.

Comment 3: Please unify the reported impedance bandwidth values for the final design. The manuscript reports slightly different lower-band bandwidth values for the final design.
For example:
• In the Abstract and Introduction, the operating range is given as 26.39–27.04 GHz, corresponding to approximately 0.648 GHz.
• In Table IV for hQ = 2 mm, the bandwidth is reported as 0.655 GHz, with range 26.38–27.04 GHz.
• In Table V for WQ = 5.5 mm, the bandwidth is reported as 0.648 GHz, with range 26.39–27.04 GHz.
The difference is small, but since these values refer to the final optimized structure, please use one consistent final value or explain why the values differ between the hQ and WQ parametric studies.

Response: Respected Reviewer, Thank you so much for this helpful comment. We are extremely sorry for the unintentional typographical mistakes. We are highly thankful to the **Respected Reviewer** for pointing out these mistakes. The impedance bandwidth of the lower operating band has been unified and consistently presented in the Abstract, Introduction,

Results and Discussion, and Conclusion sections. The reported operating range for the lower band is 26.39–27.04 GHz, corresponding to an impedance bandwidth of 0.65 GHz. This revision eliminates any ambiguity and ensures consistency in the presentation of the final optimized design results.

Comment 4: The gain statement in Section 5 and the Conclusion should be corrected In Section 5, the manuscript states:
"From measurements, a maximum gain of 8.40 dB is observed at 36 GHz." However, in the Conclusion, the manuscript states:
"The proposed MIMO radiator exhibits stable radiation patterns with a high gain of 8.40 dBi at 38 GHz…"
These two statements are inconsistent. If the measured maximum gain is observed at 36 GHz, then it should not be described as the gain at 38 GHz. Antenna gain should normally be reported in dBi, not simply dB.

Response: Respected Reviewer, Thank you so much for this helpful comment. The manuscript has been revised accordingly to ensure consistency between the gain values discussed in the measured results and the Conclusion sections. In addition, the antenna gain unit has been corrected from "dB" to "dBi", which is the appropriate unit for reporting antenna gain. The revised manuscript now consistently reports the measured maximum gain and its corresponding frequency throughout all sections of the manuscript for your kind consideration.

Comment 5: Figure 13 caption is incorrect. The caption of Figure 13 currently states:
"The comparison of isolation of the suggested 4-port MIMO antenna."
However, Figure 13 actually shows:
• gain versus frequency;
• radiation efficiency versus frequency.
Please revise the caption of Figure 13 to reflect the actual content of the figure.

Response: Respected Reviewer, Thank you so much for this helpful comment. We are extremely sorry for the typing mistaking in the caption of Figure 13. The caption has been revised to correctly reflect the presented results, which show the gain and radiation efficiency of the proposed antenna as a function of frequency, including both simulated and measured data. The revised caption is as follows: Figure 13. Comparison of radiation parameters of the presented antenna (a) Gain (dBi) and (b) Radiation efficiency (%)

Comment 6: Please distinguish radiation efficiency and total efficiency. In Section 5, the manuscript states:

"From measurements, a maximum gain of 8.40 dB is observed at 36 GHz, with corresponding total efficiency of 88.86% at 27 GHz and 75.51% at 38 GHz."
However, Figure 13(b) is labeled as radiation efficiency. Elsewhere in the manuscript, the reported simulated values are called radiation efficiencies:

• 98.81% at 27 GHz;
• 95.35% at 38 GHz.
Please clarify whether the measured values are radiation efficiency or total efficiency. If simulated values and measured values represent different efficiency definitions, this should be clearly stated.

Response: Respected Reviewer, Thank you so much for this helpful comment. We agree that the terminology used in the original manuscript may lead to confusion. The simulated and measured efficiencies presented in Figure 13(b) correspond to radiation efficiency, not total efficiency. Therefore, the statement in Section 5 referring to the measured values as "total efficiency" was incorrect and has been revised accordingly as per your valuable suggestion. Also, we have presented the accurate values of gain and radiation efficiencies at the exact resonant frequencies (26.73 GHz/38.29 GHz)

The corrected text in the revised article---

Fig. 13 highlights the gain and radiation efficiency plots for the presented 5G MIMO antenna, comparing both the simulation and experimental results. The simulated gains are 4.80/7.61 dBi, respectively at 26.73/38.29 GHz, with corresponding radiation efficiencies of 97.61% and 95.45%. The measured gains are respectively 4.63/7.22 dBi at 26.73/38.29 GHz, while the measured radiation efficiency is 94.12% at 26.73 GHz and 95.21% at 38.29 GHz. The slight discrepancies between the simulated and measured results can be attributed to fabrication tolerances, connector losses, material property variations, and measurement uncertainties.

Comment 7: Please summarize simulated and measured results in a table.
Figures 11–15 provide important measured/simulated comparisons, but the numerical values are difficult to extract precisely from plots.

Response: Respected Reviewer, Thank you so much for your valuable. A new summary table has been added to the revised manuscript, presenting the key simulated and measured performance parameters of the proposed four-port MIMO antenna, including the resonant frequencies, −10 dB impedance bandwidths, reflection coefficient, isolation, gain, radiation efficiency, ECC, diversity gain (DG), TARC, MEG, channel capacity loss, and multiplexing efficiency. This table facilitates a clear quantitative comparison between the simulated and measured results and improves the readability of the manuscript.

Comment 8: DGS trapezoid parameters should be defined more clearly. In Figure 5(c), the trapezoidal DGS section is labeled with hQ, WQ, and HQ. However, in Section 4.5, the title is :“Effect of the height (WQ) of the DGS trapezoidal”
From Figure 5(c), WQ appears to be the horizontal base or width, while HQ appears to be a vertical dimension. Please check and correct the terminology. If WQ is a width rather than a height, the section title and text should be revised.

Response: Respected Reviewer, Thank you so much for this helpful comment. "The height (W_Q) of the DGS trapezoidal" is corrected to "the horizontal base (W_Q) of the DGS trapezoidal". We apologize for the inconveniences caused to you due to typographical errors.

Comment 9: Data availability statement should be revised. The manuscript states:
"All relevant data are available within the paper."
However, the submission information also states:
"No datasets were generated or analysed during the current study."
This is not consistent with the content of the manuscript, because this work generated simulation and measurement datasets.

Response: Respected Reviewer, Thank you so much for this helpful comment. We have corrected and maintained the same data availability statement in the manuscript and in the online submission information.

Comment 10: Please check substrate material parameters. The manuscript states in Section 4.1 that Rogers RO4003C has a loss tangent of 0.025. Please verify this value carefully, because it strongly affects efficiency, bandwidth, and gain.

Response: Respected Reviewer, Thank you so much for this helpful comment. Thank you for pointing this out. We are really sorry for this typing mistake. The loss tangent is 0.0025. We have corrected it. Thank you so much.

Comment 11: Terminology and typographical corrections. The manuscript is understandable, but the following specific wording and formatting issues should be corrected:
• "Keys words" should be changed to "Keywords".
• "Valued (mm)" in Table II should be changed to "Values (mm)".
• "defective ground structure" should be written consistently as "defected ground structure".
• In Figure 16 caption, "p38 GHz" should be corrected to "38 GHz".
• "two perpendiculars patch elements" should be corrected to "two perpendicular patch elements".
• In Figure 4 caption, "different propositions" should be replaced by "different configurations" or "different arrangements".
• In the author contribution section, "Investigation, Investigation" is repeated for Sudipta Das. Please remove the duplicated word.

Response: Respected Reviewer, Thank you so much for this helpful comment. We have corrected all suggested changes in the revised manuscript for your kind consideration.

Finally, we hope that these revisions adequately address the concerns raised by Respected Reviewer 1 and improve the clarity and rigor of the manuscript.

---xx---

Reviewer 2

Comments to the Author

The manuscript presents a compact flower-shaped dual-band four-port MIMO antenna with a hybrid defective ground structure for 27/38 GHz 5G millimeter-wave applications. The topic is relevant, and the authors provide both simulation and measurement results. However, the manuscript still requires substantial improvement in terms of novelty justification, technical depth, measurement analysis, consistency of reported results, and language quality. My detailed comments are as follows：

Response to Respected Reviewer 1

We would like to express our sincere gratitude to the **Respected Reviewer 2** for the time and effort devoted to evaluating our manuscript, as well as for the constructive and encouraging comments provided. We are pleased that the reviewer found the proposed topic relevant, and mentioned about experimental validation of the work. The remarks have helped us improve several important aspects of the paper. We have carefully revised the manuscript and provided detailed responses to each comment. A detailed point-by-point response is provided below, and all modifications related to **Respected Reviewer 2's** comments are marked in blue in the revised manuscript.

Comment 1: The claimed novelty of the flower-shaped radiator and hybrid DGS is not sufficiently distinguished from existing DGS-based MIMO antennas. The authors should clearly explain the structural and electromagnetic differences from prior works.

Response: Respected Reviewer, Thank you so much for this helpful comment. The novelty of the proposed work lies in the combination of a flower-shaped radiator geometry with a hybrid defected ground structure (DGS) specifically optimized for dual band mmWave 5G applications. The flower-shaped patch elements are strategically placed in orthogonal arrangements and the suggested hybrid DGS consists of a central circular slot combined with four surrounding trapezoidal cut-outs to provide improved performance. The proposed antenna partially operates within 5G NR mmWave n258/n260 bands and is suitable for 5G Fixed Wireless Access (FWA) applications.

Unlike conventional DGS-based MIMO antennas reported in the literature, which typically employ simple rectangular, circular, or slot-type ground defects, the proposed hybrid DGS introduces a combined structural modification that simultaneously enhances impedance matching, improves port isolation, and contributes to antenna miniaturization.

In addition, the flower-shaped radiator provides improved current distribution and increased effective electrical length, which enables compact dual-band operation while maintaining high gain and radiation efficiency. The integration of this radiator with the optimized hybrid DGS results in superior overall performance in terms of bandwidth, isolation, and radiation characteristics compared to existing designs. Furthermore, the proposed four-port MIMO

radiator provides excellent diversity metrics, ensuring improved signal reliability and maximized data throughput.

- ❖ **The following revised text related to novelty is included in the introduction section of the revised manuscript for your kind consideration.**

The novel contribution of this study is the development of a four-port MIMO antenna that combines four identical orthogonally arranged flower-shaped elliptical patch elements with a specifically engineered and systematically optimized hybrid defected ground structure (DGS) to achieve enhanced isolation across two distinct mmWave operating bands. The proposed DGS configuration is uniquely designed by a central circular slot combined with four surrounding trapezoidal slots to effectively improve isolation between the radiating patches while preserving high gain and radiation efficiency performance. Furthermore, it offers a favorable combination of dual-resonant band functionality, high isolation, size miniaturization, reliable radiative performance as well as excellent diversity metrics, ensuring improved signal reliability and maximized data throughput. This integrated design approach distinguishes the proposed antenna from previously reported MIMO structures, making it a potential choice for dual band 5G mmWave communication applications.

The following point summarizes the novel aspects of the suggested work:

- **Compact size:** The four identical elliptical monopole elements, transformed into flower shaped with a novel hybrid defected ground plane occupies an area of only 21 mm × 21 mm (1.849 λ×1.849 λ).
- **Hybrid DGS configuration:** A distinctive DGS, consisting of circular and trapezoidal shapes are strategically incorporated at the ground plane's base to minimize mutual coupling, improving overall isolation. This helps minimize the electromagnetic interference, improving overall system reliability.
- **Dual-Resonance and stable radiation performance:** The designed four port MIMO radiator operates from 26.39-27.04 GHz and 38.11-38.47 GHz, partially covering the spectrums of 5G NR n258/n260 bands, yielding stable gains of 4.80 and 7.61 dBi at 26.73 and 38.29 GHz, respectively along with radiation efficiencies of 97.61% and 95.45%, respectively.
- **Optimized metrics:** Exceptionally low ECC, high DG, competitive CCL, MEG, TARC and desired multiplexing efficiency confirm its superior MIMO diversity performance.
- **Performance validation and applicability:** All the analyzed results obtained by using CST MWS tool are validated through measurements of the realized antenna prototype. The measurement results confirm its dual band utilization. The proposed antenna partially operates within 5G NR mmWave n258/n260 bands and is suitable for 5G Fixed Wireless Access (FWA) applications.

Comment 2: The effects of R_{DGS}, h_Q, and W_Q are studied, but the final parameter selection lacks a clear optimization criterion. The authors should explain the trade-off among bandwidth, isolation, gain, efficiency, and resonance accuracy.

Response: Respected Reviewer, Thank you so much for this helpful comment. The final parameter selection was not based on a single performance metric, but rather on a comprehensive trade-off among impedance bandwidth, isolation, gain, radiation efficiency, and the accuracy of the resonant frequencies at the target bands. During the parametric studies, each parameter exhibited different and sometimes competing effects on the antenna performance. For instance, improvements in impedance matching could slightly affect isolation, while variations in the DGS radius influenced both bandwidth and gain.

Therefore, the final optimized values were chosen to achieve the best overall balance between these performance indicators, ensuring acceptable impedance matching, high isolation, stable gain, high radiation efficiency, and accurate resonance alignment with the desired operating frequencies. Kindly consider, please.

Comment 3: The measured efficiency is much lower than the simulated efficiency, especially at 38 GHz. The authors should discuss possible reasons such as fabrication tolerance, connector loss, soldering effects, substrate deviation, and measurement uncertainty.

Response: Respected Reviewer, Thank you so much for this helpful comment. We would like to clarify that the simulated and measured results reported in Fig. 13 show a strong agreement in terms of overall trends and antenna performance behavior. Although some numerical differences exist between simulation and measurement, these variations are expected due to practical implementation effects.

The simulated results represent an ideal electromagnetic model, while the measured results corresponding to the fabricated prototype are possibly affected by fabrication tolerances, substrate's dielectric-property variations, copper roughness, etching inaccuracies, SMA connector soldering effects, cable and connector losses, antenna alignment errors, residual reflections in the anechoic chamber. However, the measured results remain in acceptable agreement with the simulations and confirm the dual band operating mechanism and the effectiveness of the proposed antenna design.

Therefore, the observed differences are within an acceptable range and do not affect the validity of the proposed work. As discussed in the manuscript (Fig. 13), the simulated results show a radiation efficiency of about 97.61% at 26.73 GHz and 95.45% at 38.29 GHz and from measurements, the radiation efficiency is about 94.12% at 26.73 GHz and 95.21% at 38.29 GHz.

Comment 4: Some reported values of reflection coefficient, bandwidth, gain, and efficiency appear inconsistent across the abstract, main text, tables, and conclusion. The authors should carefully verify all numerical data.

Response: Respected Reviewer, Thank you so much for this helpful comment. We sincerely thank the reviewer for this important observation. Following the reviewer's comments, a comprehensive verification of all reported numerical results has been performed throughout the manuscript. Particular attention was given to the reflection coefficient, impedance bandwidth, gain, and efficiency values reported in the abstract, main text, tables, figures, and Conclusion. Any inconsistencies identified during this review process have been corrected, and the numerical values have been updated to ensure complete consistency across all sections of the manuscript for your kind consideration.

Please refer to our responses to Reviewer 1's comments, where the corresponding corrections are described in detail.

Comment 5: Only ECC and DG are discussed. Additional metrics such as TARC, MEG, channel capacity loss, and multiplexing efficiency should be included to better evaluate the four-port MIMO performance.

Response: Respected Reviewer, Thank you so much for this helpful comment. We sincerely thank the reviewer for this valuable suggestion. In response to this comment, the MIMO performance analysis has been significantly expanded. The revised manuscript includes the additional MIMO performance metrics, including the Total Active Reflection Coefficient (TARC), Mean Effective Gain (MEG), Channel Capacity Loss (CCL), and Multiplexing Efficiency (ME), in addition to the previously discussed Envelope Correlation Coefficient (ECC) and Diversity Gain (DG) for your kind consideration. Furthermore, a new table (Table VI) has been included to present a comprehensive comparison between the simulated and measured values of all these MIMO performance parameters. The obtained results are thoroughly discussed, demonstrating a close agreement between simulation and measurement and confirming the excellent diversity and multiplexing performance of the proposed four-port MIMO antenna system.

Comment 6: Table VI is useful but not fully fair, since the compared antennas differ in size, number of ports, bandwidth, substrate, and measurement conditions. More normalized and complete comparison indicators should be added.

Response: Respected Reviewer, Thank you so much for this helpful comment. We agree that a completely fair comparison among different MIMO antenna designs is challenging because the reported antennas in the literature differ in several aspects, including the number of ports, physical dimensions, substrate materials, operating bandwidths, and measurement conditions. The purpose of Table VI (Table VII in the new version) is to provide a general performance comparison with representative state-of-the-art designs operating in similar frequency ranges. We acknowledge that direct one-to-one comparisons are inherently limited due to the differences in results of the reported designs. However, we have revised the table by additionally including details of designed methodology electrical size, number of ports used in antenna configurations, gain-to-area ratio, and various MIMO diversity parameters.

Comment 7: The manuscript mainly presents simulation curves but does not adequately explain why the proposed patch generates dual-band resonance or how the DGS suppresses mutual coupling. More discussion based on surface current distribution or equivalent current paths is needed.

Response: Respected Reviewer, Thank you so much for this helpful comment. The dual-band resonance observed in the proposed antenna is mainly attributed to the combination of the flower-shaped radiator geometry and the modified current paths introduced by the structure. The irregular shape of the patch creates multiple effective current paths with different electrical lengths, which support two distinct resonant modes corresponding to the operating bands at 26.73 GHz and 38.29 GHz. Regarding the mutual coupling reduction, the incorporation of the defected ground structure (DGS) plays a key role in suppressing surface wave propagation and disturbing the current flow on the ground plane. This results in reduced coupling between adjacent elements by increasing the effective current path length and creating a filtering effect that improves isolation.

Detailed explanations:

Dual-Band Resonance Mechanism (Based on Surface Current Distribution)

The proposed patch antenna achieves its dual-band resonance behavior at 26.73 GHz and 38.29 GHz by utilizing different current path distributions across its asymmetric, multi-featured geometry.

At lower band resonance, the surface currents are primarily concentrated along the full length of the patch radiator and its main feed line. The effective current path length corresponds roughly to a half-wavelength at 26.73 GHz, encompassing the entire length from the feed point to the top curvilinear shapes of the flower-shaped patch. When Port 1 is excited at lower resonance, high-intensity current densities (A/m) are observable over the entire body of the excited patch element. At 38.29 GHz, the current redistributes itself to flow dominantly through the smaller geometric sub-structures, specifically the comb-like slots/teeth variations. The higher frequency excites a higher-order mode or shorter localized paths. As seen in Figure 16 (c) or (e), at higher frequencies, the current density hot-spots shift towards the edges, slots, and inner junctions of the patch, validating that these specific sub-wavelength structural variations are responsible for the second resonance.

Mutual Coupling Suppression via Defected Ground Structure (DGS)

The DGS (trapezoidal section) acts as a decoupling barrier that intercepts and block surface waves between adjacent ports. For lower band operation, when Port 1 is excited [Figure 16 (a)], the current is restricted mostly to the active element. The DGS slot suppresses surface wave propagation across the ground plane. Consequently, Figure 16 (a) shows near-zero or extremely weak current distribution on the unexcited adjacent patches (Port 2, Port 3, Port 4), confirming low mutual coupling. At 38.29 GHz, surface waves traveling through the common substrate are trapped by the specific trapezoidal DGS profile. The slot dimensions create an out-of-phase reflection or act as a high-impedance surface for the surface currents at upper resonance. This

stops electromagnetic energy from leaking into neighbouring ports, providing required satisfactory levels of isolations.

- ❖ **To further support this explanation, the following summarized discussion based on surface current distribution and role of DGS has been added to the revised manuscript.**

To understand the physical insight behind the dual-band operation and isolation enhancement, the surface current distributions at 26.73 GHz and 38.29 GHz are analyzed in Figure 16. At 26.73 GHz [Figure 16 (a)], the surface current flows uniformly along the entire length of the patch, signifying that the fundamental mode governs the lower band. Conversely, at 38.29 GHz, intense current concentration shifts towards the slot edges and curvilinear discontinuities, generating a secondary shorter effective path that yields the upper resonant band.

Furthermore, the decoupling mechanism introduced by the trapezoidal-shaped based defected ground structure (DGS) is apparent in minimizing mutual coupling among orthogonally placed antenna elements. When an individual port is active, the DGS behaves as a high-impedance band-stop wall. It traps and perturbs the ground plane currents, preventing them from coupling into adjacent elements. This causes the unexcited patches to exhibit minimum current intensity, which directly supports the highly suppressed mutual coupling observed in the S-parameter characteristics.

Finally, we hope that these revisions adequately address the concerns raised by Reviewer 2 and improve the clarity and rigor of the manuscript.

----------* Respected Editors and Reviewers, Thank You so much once again for your valuable comments on the improvement of the article--------*****
